# A Quantitative Examination of Catalyst Component Impacts on Hydrogen Adsorption and Spillover

**DOI:** 10.1021/jacs.6c08946

**Published:** 2026-08-04

**Authors:** Audrey M. Battiste, Tae Yong Yun, Kelle D. Hart, Rebekah J. Snellings, Mohammad Hamidizirasefi, Angela P. Abraham, Bert D. Chandler

**Affiliations:** † Department of Chemistry, 8082 The Pennsylvania State University, University Park, Pennsylvania 16802, United States; ‡ Department of Chemical Engineering, 52469 The Pennsylvania State University, University Park, Pennsylvania 16802, United States

## Abstract

Hydrogen spillover is a widely recognized but poorly understood surface phenomenon. We coupled volumetric chemisorption with Fourier transform infrared spectroscopy, thermogravimetric analysis, and acid–base titrations to quantify the roles of metal (Pt, Au) and support chemistry (anatase, rutile, P25 titania) on spillover. We show that metal identity has little to no impact on the amount of spillover hydrogen. Spillover pressure dependence is essentially the same on Au/TiO_2_ and Pt/TiO_2_, indicating H_2_ adsorption is equilibrated across both the metal and support. Conversely, spillover is highly sensitive to support surface chemistry, as rutile TiO_2_ stabilizes ∼5× more surface hydrogen than anatase TiO_2_. This change is due to a combination of factors, including the rutile surface’s higher proton affinity, stabilized surface electronic states, and larger surface entropy. This work highlights the utility of Au/MO_x_ catalysts as control materials for spillover, as they enable quantitative evaluation of H_2_ adsorption on active metals and spillover onto the support.

## Graphical abstract

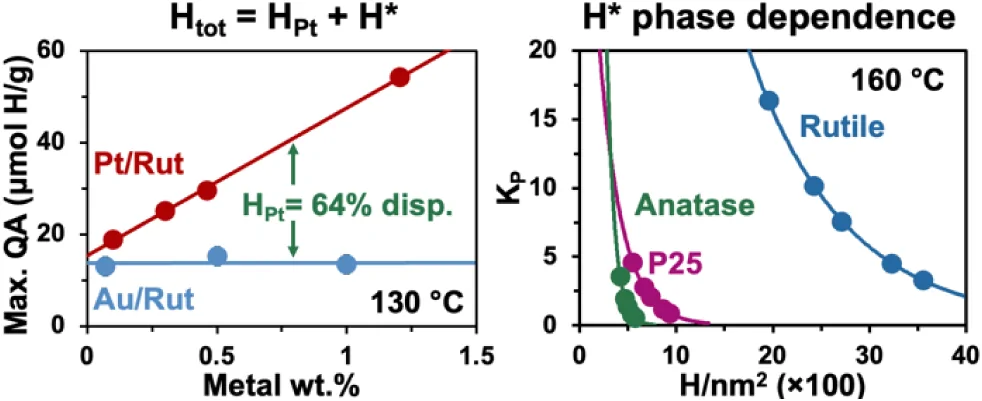

## Introduction

Catalytic processes such as selective hydrogenations,^1^ CO oxidation,^2^ the water-gas shift reaction,^3^ hydro-deoxygenation,^4,5^ and hydrogen production/storage^6^ rely on controlling hydrogen surface chemistries. This includes “hydrogen spillover”, a term used to describe a combination of mobile hydrogen species and support reduction events. Spillover is most prominent on reducible oxides (e.g., titania, ceria) which have important applications in photocatalysis, semiconductor research, and energy storage.^7–10^ However, there is little quantitative data to support the conclusions regarding how and why various surface chemistries foster mobile hydrogen.^11^

Spillover rapidly transports H atoms over μm distances, offering unique H-transport and catalysis chemistries.^12^ Spillover is difficult to study because it is difficult to distinguish transient mobile H from support reduction.^13^ We recently developed methods for quantifying reversibly adsorbed H* on Au/TiO_2_ catalysts, showing spillover is driven by the surface entropy of both H* and the support.^14–18^ The support surface chemistry plays a key role in spillover;^16–18^ understanding this role is key to controlling H*.

Spillover from active metals (Pt, Pd, Ru) has greater practical importance in catalysis and energy storage.^19^ However, deconvoluting H* from H adsorbed on metal sites (M-H) is difficult, often precluding quantitative H* studies on these catalysts.^20–22^ In this work, we examine H_2_ adsorption on γ-Al_2_O_3_ and TiO_2_ supported Au and Pt catalysts to address these two fundamental spillover surface chemistry issues. This allows us to discriminate between M-H and H* adsorption, showing spillover is essentially independent of the metal identity. We also show large support phase effects, both in terms of the amount of adsorbed H and in thermodynamic values extracted from van’t Hoff analyses. Rutile facilitates a > 5× increase in surface H* concentration relative to anatase, largely due to the significantly larger adsorption equilibrium constant value and more favorable adsorption thermodynamics. These results significantly advance our understanding of spillover.

### Spillover Definitions

Active metals (e.g., Pt, Pd) initially adsorb hydrogen on metal sites (M-H); however, H-atom equivalents (a proton and an electron) can be transferred to reducible oxides as hydrogen spillover, H*.^23^ The “spillover” term was initially coined by Vannice and Boudart^24^ to describe support reduction associated with the conversion of WO_3_ to WO_2_:

1 $${\mathrm{WO}}_{3}+H_{2}\to {\mathrm{WO}}_{2}+H_{2}O$$

While this reaction is associated with the original description of spillover,^16–18^ definitions have since been refined. Specifically, classic reviews by Connor and Falconer,^25^ and more recently Prins,^16^ recognized two separate processes are involved, as did early work from Boudart.^20,24,26^ The most easily observed process is the reaction between H atom equivalents and reducible metal oxides. This chemical reaction stores electrons in the support as reduced cations and/or O vacancies, releasing the protons as water. Because this irreversible reaction completely decouples the protons and electrons, they concluded it is best described as support reduction. Support reduction must be enabled by highly mobile H-atoms (H*);^26^ we use Prins’ definition of spillover as highly mobile H-atom equivalents to distinguish spillover from support reduction.

There are at least two types of spillover consistent with this definition. For more reducible supports such CeO_2_, Christopher’s group developed a localized bonding model.^27^ In this case, electrons are localized on Ce^3+^ cations and charge-balanced with the associated proton. No water is lost, no irreversible O vacancy is required, and protons and electrons can be transmitted via electron transfer between cerium cations.

The work described in the present manuscript deals exclusively with mobile H-atom equivalents involving delocalized electrons. These delocalized, mobile H-atom equivalents (H*) are associated with fully reversible weak H_2_ adsorption, i.e., all H_2_ desorbs when the gas phase H_2_ is removed. H* adsorption is partially driven by favorable H* surface entropy, which is the entropy of the mobile H* species.^19,20^ These properties distinguish H* from H adsorption on metal surfaces or support defect sites,^21^ making it an important phenomenon in catalysis.^22,23^

## Results and Discussion

### Catalyst Matrix and Spillover Behavior

Hydrogen adsorption on active metals (e.g., Pt, Pd) initially yields stable M-H species. However, extended Au surfaces do not activate H_2_;^28^ on Au/TiO_2_ catalysts, H_2_ is activated at the metal–support interface (MSI), initially yielding 2 H* species on MSI hydroxyls.^14,15,29–31^ We prepared four catalysts (Figure 1A) to compare combinations of active/inactive metals for M-H adsorption (Pt/Au, respectively) and active/inactive supports for spillover (rutile/γ-alumina, respectively).^32^ We refer to the “inactive” materials only to indicate they are relatively unreactive toward H* or M-H adsorption. Note, we compare several catalyst properties (metal, support surface area, etc.) throughout this work. Accordingly, individual plots will normalize adsorption data differently to best highlight a particular comparison. For completeness, data are reproduced in multiple adsorption units in the SI (Figure S7).

**Figure 1:**
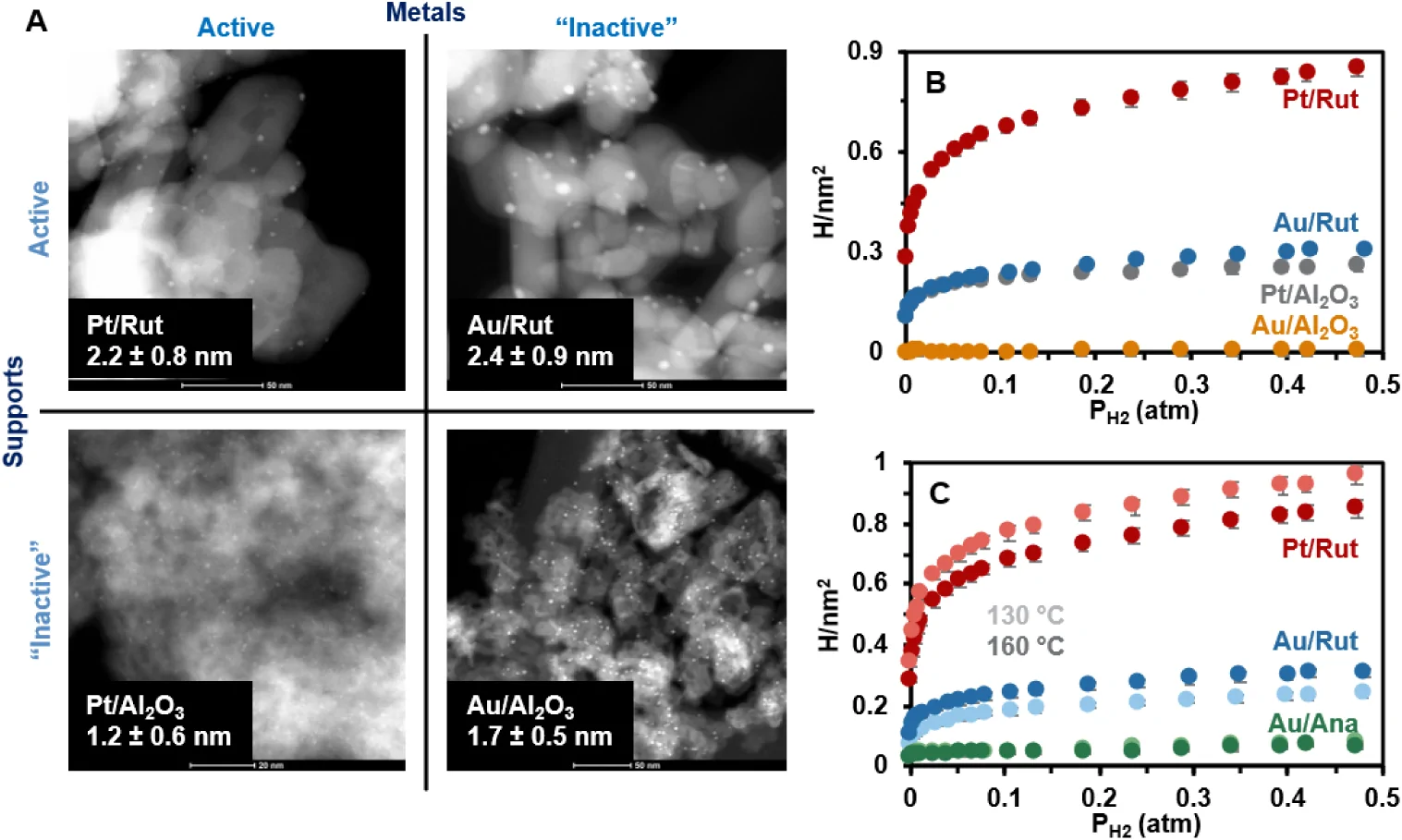
(A) STEM images of four 1% M/MO_x_ catalysts initially tested, arranged into a grid by the “activity” of both the metal and the metal oxide used. For our purposes, “active” surfaces (Pt and TiO_2_) adsorb significant quantities of H_2_ and “unreactive” surfaces (Au and γ-Al_2_O_3_) do not. (B) H_2_ adsorption isotherms collected at 160 °C on 1% Au/rut (blue), 1% Pt/γ-Al_2_O_3_ (gray), 1% Pt/rut (red), and 1% Au/γ-Al_2_O_3_ (gold) normalized to the support surface area. The amount of adsorbed H_2_ correlates with the presence of an active surface. (C) H_2_ adsorption isotherms on 1% Pt/rut (red), 1% Au/rut (blue), and 1% Au/ana at 130 °C (light colors) and 160 °C (dark colors). The H* capacity of rutile is roughly 5× larger than anatase; Pt/rut and Au/rut show opposite temperature effects for H_2_ adsorption. Error bars are the averages of the standard deviations for at least 4 isotherm measurements per catalyst.

Figure 1B shows H_2_ adsorption isotherms on the four catalysts. At these temperatures (130,160 °C) all H_2_ adsorption is reversible under vacuum (further details in the SI and Figure S7). Significant H_2_ adsorption occurs when the catalyst contains either active component (Pt or rutile). The highest surface normalized adsorption capacity occurs on Pt/rutile (Pt/rut), where both components are active. Figure 1C shows Au/rutile (Au/rut) adsorbs ∼5× more H than Au/anatase (Au/ana). This is a surprisingly large titania phase effect, as the anatase adsorption properties are more similar to alumina than rutile (see below). Both the metal and the support clearly impact H_2_ adsorption; we systematically address each constituent below.

### Effect of Metal on H_2_ Adsorption and Spillover

Hydrogen adsorption on Pt/γ-Al_2_O_3_ is consistent with adsorption on the metal. A loading study (Figure S8) showed a linear increase in H_2_ adsorption with Pt loading and a y-intercept near 0, indicating no spillover. Figure 1B shows small, but measurable H_2_ adsorption on Au/γ-Al_2_O_3_, consistent with adsorption at a fraction of the MSI sites. Extended Au surfaces do not activate H_2_,^28^ yet Au/γ-Al_2_O_3_ is an active hydrogenation catalyst;^29,33–35^ H_2_ adsorption at a small fraction of interface sites is consistent with these reports. Both the Pt and Au data are consistent with the conclusion there is little to no spillover on alumina, as expected.^12^

While total adsorption quantities differ, the Au/rut and Pt/rut isotherms have nearly identical shapes (Figure 2A). Note the Pt/rut isotherm does not fit a Langmuir isotherm, which is also consistent with spillover playing an important role in the adsorption.^16–18^ This has several important consequences. First, it indicates the equilibrium constant for H adsorption is independent of the metal (T_ads_ = 130 °C). This is surprising, and suggests the adsorption is essentially additive between the Pt and rutile components for Pt/rut.

**Figure 2:**
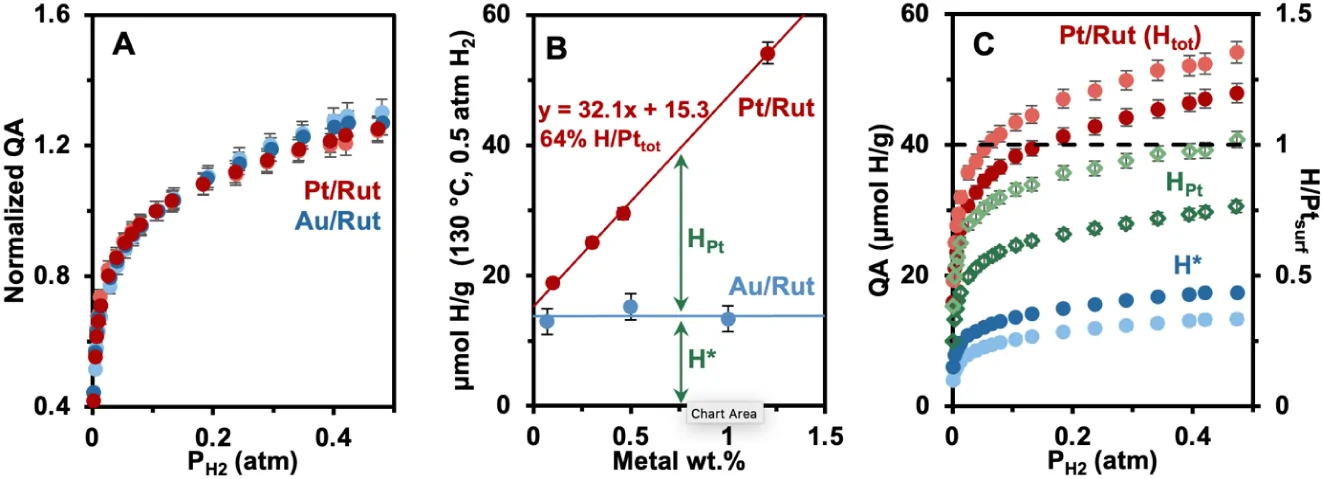
(A) H_2_ adsorption isotherms on 1% Pt and Au /rut at 130 °C (light colors) and 160 °C (dark colors) normalized such that QA = 1 at P_H2_ = 0.1 atm to reflect more subtle variations in isotherm shape. (B) H_2_ adsorption quantity (0.5 atm H_2_, 130 °C) vs metal wt.% of several Au/rut and Pt/rut catalysts (0.07–3.5% Au/rut, 0.1–1.0% Pt/rut). Error bars are the averages of the standard deviations for at least 4 isotherm measurements per catalyst. (C) H_2_ adsorption isotherms at 130 °C (light colors) and 160 °C (dark colors) on 1% Pt/rut, 1% Au/rut (labeled as H*), and H_2_ adsorption on Pt calculated by subtracting the QA values of Au/rut from Pt/rut at each pressure (green diamonds labeled as H_Pt_) plotted as μmol H*/g (left axis) and H/Pt_surf_ (right axis).

We examined this through metal loading studies. Figure 2B plots H adsorption at 0.5 atm H_2_, 130 °C for Pt/rut and Au/rut. The study shows H_2_ adsorption on Au/rut is independent of the Au loading. This is fully consistent with adsorption as H* and is inconsistent with H adsorption on Au surfaces, as we have previously shown.^15–17^

Hydrogen adsorption on the Pt/rut catalysts shows a linear dependence on the Pt loading, consistent with H adsorption on Pt. However, the data has a significant y-intercept, which can be interpreted as the spillover at this P_H2_ and temperature. The y-intercept of the Pt/rut data (15 μmol H/g) is nearly the same as the average H* surface concentration on the Au/rut catalysts (14 μmol H/g). Moreover, the slope yields a H/Pt dispersion of 64%, which is in excellent agreement with the average dispersion of these catalysts measured by STEM (66%, see SI).

Based on this internally consistent quantitative adsorption data, it is possible to differentiate between H-Pt and H* simply by comparing the Pt/rut and Au/rut catalysts. In other words, one can treat the Au/rut isotherm as the spillover portion of the Pt/rut isotherm and extract the H-Pt portion simply by subtracting the two (eq 1). Figure 2C shows this equation is consistent at sufficiently low temperatures. However, at elevated temperatures, H-Pt is likely below unity due to entropically unfavorable nature of adsorption on Pt.

2 $$H_{\mathrm{tot}}\left(\mathrm{Pt}/\mathrm{rut}\right)=H_{\mathrm{Pt}}+H^{*}=H_{\mathrm{Pt}}+H_{\mathrm{tot}\;}\left(\mathrm{Au}/\mathrm{rut}\right)$$

Equation 2 also allows us to evaluate the H speciation for Pt on anatase and P25, simply by subtracting the Au data from the Pt data on the same support. For both anatase and P25, this results in a H/Pt_surf_ ≈ 1 (SI, Figure S8C–D), consistent with the rutile data.

Another consequence of the comparison in Figure 2A is the surprising finding that the equilibrium constant for H-Pt and H* is essentially the same at a given P_H2_. This is likely a consequence of the rapid exchange between H-Pt and H*. Provided the exchange is fast, the equilibrated catalyst surface has a single chemical potential, giving rise to a single K_P_ value describing both Pt-H and H* adsorption. Indeed, this is the core challenge to distinguishing between the two types of adsorption. The strong enthalpic driving force for Pt-H adsorption (ΔH_ads_ ≈ −100 kJ/mol)^36–38^ coincides with a strong entropic driving force for H* adsorption at low coverages.^16–18^ Thus, both types of adsorbed hydrogen (Pt-H and H*) contribute across the entire pressure range measured, although not necessarily equally at all pressures (i.e., enthalpically favorable adsorption likely dominates at low pressures). The transition between these two limiting cases is more gradual than we appreciated.

The opposite temperature dependence for H_2_ adsorption on Pt/rut and Au/rut (Figure 1C) is easily rationalized with this understanding. Adsorption on Au/rut increases with temperature due to the entropic driving force for spillover. The extracted H_Pt_ isotherms (Figure 2C) show Pt-H coverage decreases at higher temperatures, as expected for enthalpically driven Pt-H adsorption. The increased H* on Pt/rut cannot compensate for the lower Pt-H coverage, resulting in less total adsorption at higher temperature.

More broadly, this provides a simple and rigorous method for differentiating spillover from adsorption on metals in other systems. Proper comparisons with Au/MO_x_ catalysts can provide quantitative evaluations of how spillover surface concentrations change with temperature and pressure. These metrics are critical for understanding reaction kinetics in systems where spillover may play a role in catalytic activity.

### TiO_2_ Phase Effects on H_2_ Adsorption

Figure 3A shows adsorption isotherms reported in typical units (μmol H/g_cat_). Hydrogen adsorption on Au/alumina shows three distinct characteristics: (i) the isotherm saturates at very low pressure; (ii) total adsorption is less than half the number of MSI sites (SI Figure S7); and (iii) total adsorption decreases as temperature increases (Figure 3B). This is consistent with a small amount of enthalpically driven H_2_ adsorption occurring at a fraction of the MSI sites (roughly 1/3). We cannot rule out the possibility that adsorption occurs on a small number of highly undercoordinated Au sites, but this would require a significant shift in the underlying chemistry. Hydrogen adsorption on Au/anatase shares all three of these characteristics; in both cases this indicates enthalpically driven adsorption at the interface with very little spillover, which is driven by entropy.^16–18^

**Figure 3:**
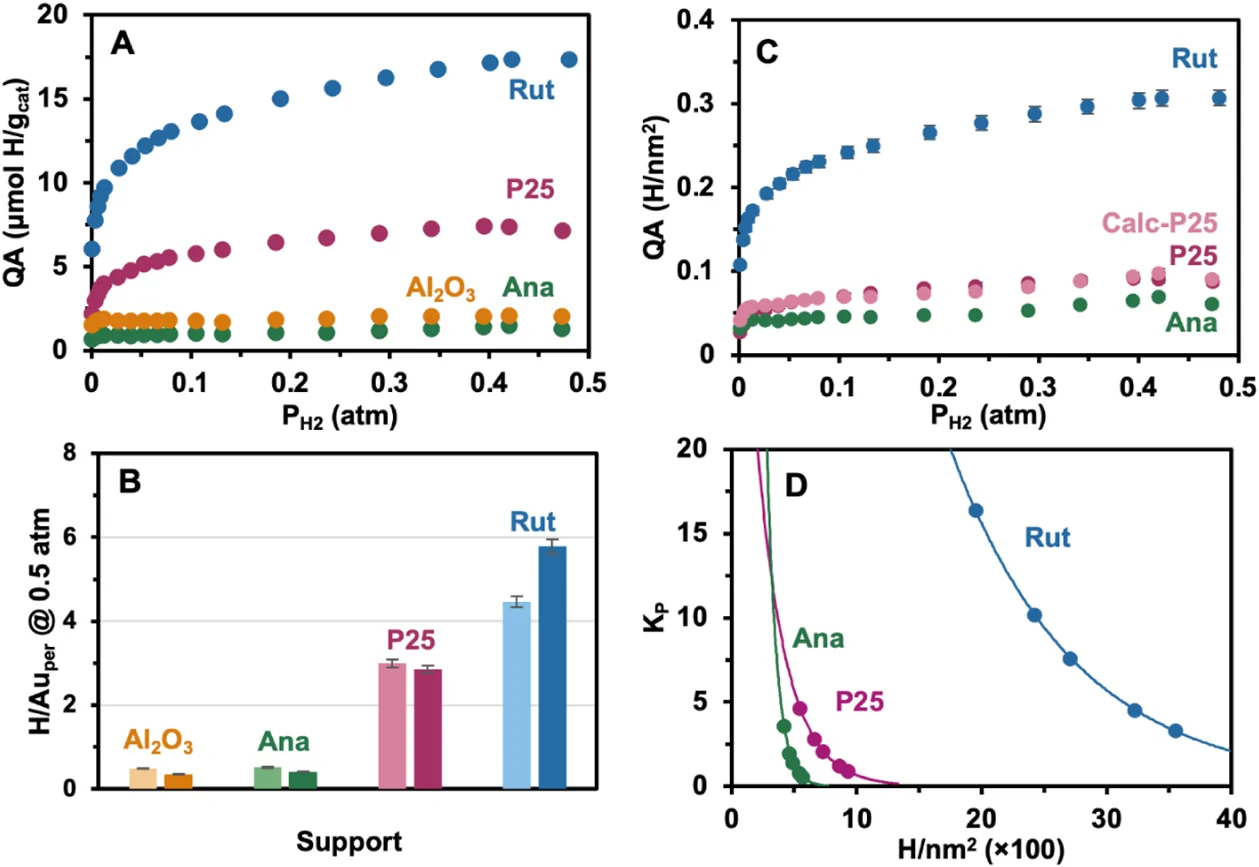
(A) Volumetric H_2_ adsorption isotherms on 1% Au/MO_x_ catalysts at 160 °C. (B) Total H_2_ adsorption normalized to the Au perimeter sites (H/Au_per_) at P_H2_ = 0.5 atm and 130 °C (light colors) or 160 °C (dark colors); all catalysts are 1% Au by weight. (C) H_2_ adsorption isotherms on 1% Au/TiO_2_ catalysts at 160 °C; the Calc-P25 isotherm sums normalized isotherms for anatase and rutile (88% ana, 12% rut). (D) Adsorption equilibrium constants vs H* coverage at 160 °C. All error bars are standard deviations of at least 3 different measurements.

In contrast, Au/rutile and Au/P25 show (i) steady increases in adsorption with pressure; (ii) total adsorption significantly exceeds the number of MSI sites (SI Figure S7); and (iii) H_2_ adsorption on Au/rut increases with temperature. These are hallmarks of entropically favorable adsorption which characterizes spillover.^16–18^ Figure 3C normalizes the adsorption to the support surface area to show spillover as a surface concentration instead of a gravimetric quantity. Small increases in adsorption at higher pressure suggest a small amount of spillover on anatase. A calculated P25 isotherm based on the P25 phase composition (Figure 3C, details in SI) is identical to the experimental isotherm. This indicates there is no significant impact of the grain boundaries and H_2_ adsorption on P25 is dominated by spillover onto rutile.

Figure 3D shows the coverage dependence in adsorption equilibrium constants (K_p_), determined at 160 °C as previously described.^16–18^ Extracted K_p_ values change dramatically with H* coverage due to the strong influences of the H* translational entropy.^16–18^ These data similarly show spillover is considerably more favorable on rutile than anatase.

Constant coverage van’t Hoff studies (Figure 4A–C) provide insight into the origins of the phase effects on spillover. These data must be interpreted carefully as the extracted ΔH and ΔS values are apparent (ΔH_app_ and ΔS_app_), not intrinsic (ΔH_ads_ and ΔS_ads_) values.^16–18^ Fundamentally, van’t Hoff studies evaluate temperature dependent changes in equilibrium constants. In H* adsorption, two sets of equilibria simultaneously change: (i) the H adsorption equilibrium between H* and the surface and (ii) the complex equilibria describing proton exchange across the TiO_2_ surface. Van’t Hoff studies measure the temperature effects on both sets of equilibria; because the surface hydroxyls far outnumber H*, the apparent values are heavily weighted toward changes to the surface equilibria. This makes it exceedingly difficult to determine intrinsic thermodynamic values.

**Figure 4:**
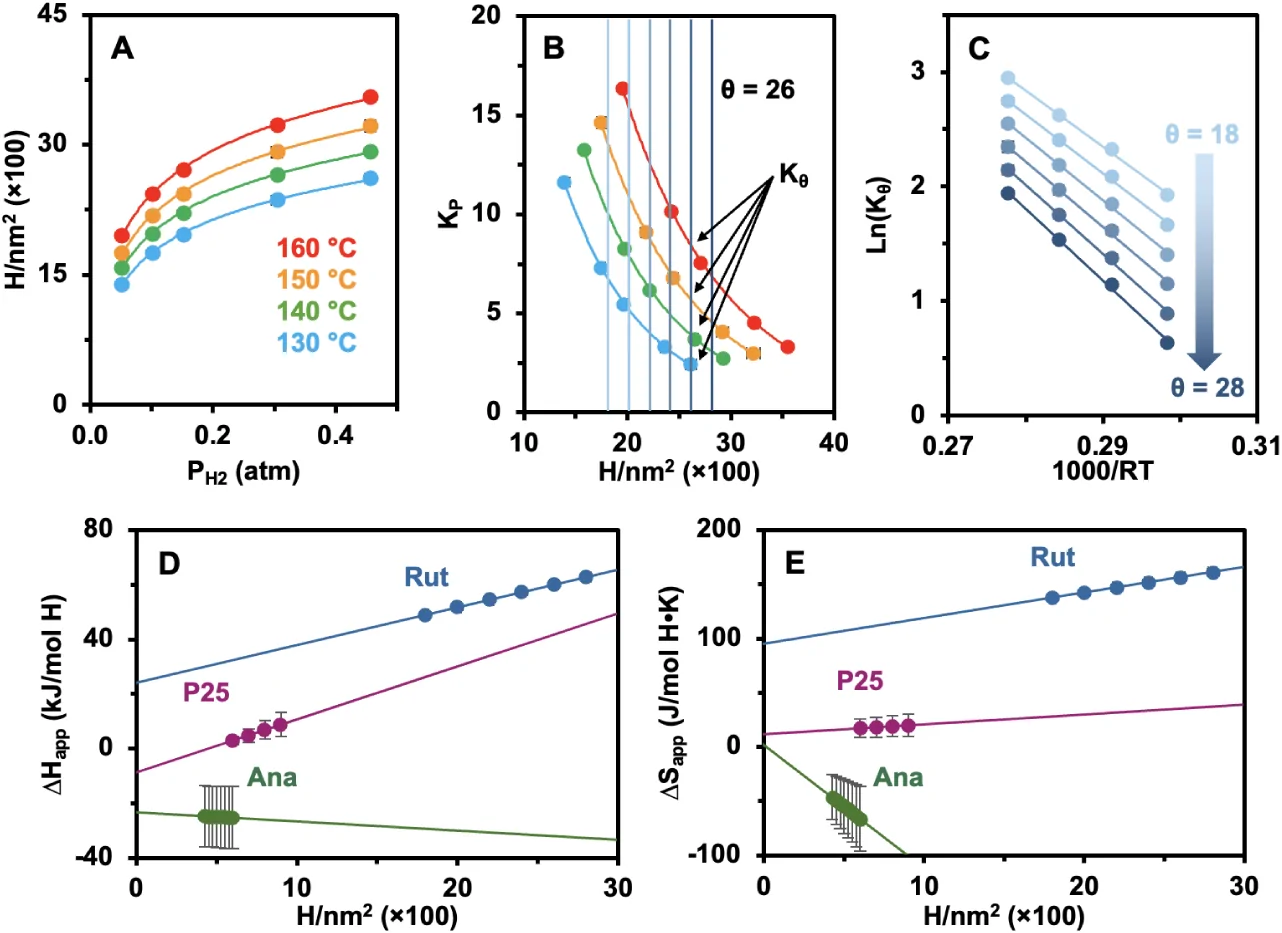
(A) H_2_ adsorption isotherms on 1% Au/rut collected via in situ FTIR spectroscopy at several temperatures. (B) Extracted K_P_ values plotted vs H coverage for the four temperatures. The vertical lines correspond to arbitrary choices for H* coverages; (C) Van’t Hoff plot for H_2_ adsorption on Au/rut at several H* coverages. Each K_θ_ value was determined from the intersection between the fitted curve and the corresponding vertical line panel B. Plots of apparent ΔH (D) and ΔS (E) values vs coverage for H_2_ adsorption on 1% Au on rutile (blue), P25 (purple), and anatase (green). All error bars are standard deviations of at least 3 different measurements. Uncertainties in (D) and (E) include standard errors from the van’t Hoff plot linear regression.

The ΔH_app_ and ΔS_app_ values are linear with H* coverage (θ_H*_), (Figure 4D–E; Table 1) so examining thermodynamic parameters extrapolated θ_H*_ = 0 provides a consistent reference point to compare catalysts with different H* coverages. This reduces, but does not eliminate, the influences of surface entropy on the extracted parameters (details in the SI, especially in Figure S18 and eq S15). For Au/anatase, individual ΔH_app_ values showed little temperature dependence, and the ΔS_app_ value at θ_H*_ = 0 was approximately 0 J/mol·K. This is consistent with enthalpically driven adsorption primarily at the interface, where electron donation from Au enriches the MSI hydroxyls.^15^ The thermodynamic data therefore suggest relatively little spillover is present on anatase under these conditions. The ΔH_app_ value for H* adsorption on anatase (−25 kJ/mol) is consistent with weak H_2_ adsorption at the MSI; at present, this is our best estimate for the intrinsic adsorption enthalpy for H* on anatase.

**Table 1:** K_P_ Values Measured at 160 °C; ΔH_0, app_, and ΔS_0, app_ Calculated from Van’t Hoff Analysis (Figure 3G–I)^a^

	Au/Rut	Au/P25	Au/Ana
**K**_**P**_ **range at 160 °C**	3.3–16.4	0.9–4.6	0.5–3.5
**ΔH**_**0, app**_ **(kJ/mol H)**	24.2 ± 0.4	–9 ± 5	–23 ± 10
**ΔS**_**0, app**_ **(J/mol H·K)**	95 ± 1	12 ± 4	2 ± 1

a All uncertainties are standard deviations from at least 3 samples.

On rutile, the van’t Hoff studies yield large positive ΔH_app_ and ΔS_app_ values at 0 H* coverage. These values are physically unrealistic, and indicate surface proton exchange equilibria dominate the changes evaluated by the van’t Hoff study.^17,18^ It is not presently possible to deconvolute the intrinsic H* adsorption equilibrium constant from the surface entropy contributions based on this data. However, this suggests facile proton exchange between surface hydroxyls may be an important driver of the higher H* adsorption on rutile.

The values for P25 are readily interpreted as a blend of anatase and rutile. Both ΔH_app_ and ΔS_app_ increase somewhat with H* coverage, indicating an important role of the titania surface equilibria. This is consistent with the role of rutile surface sites in fostering spillover.

### Origins of TiO_2_ Phase Effects

#### Bulk Band Gap

We considered how the support chemistry might impact the ability of the materials to stabilize loosely coupled H^+^ and e^–^ pairs. Diffuse reflectance (DR) UV–visible spectra, expressed as Tauc plots (Figure 5A)^39^ show relatively little variation and no trend among the bulk band gaps (the x-intercept of the linear portion of each plot). In particular, P25 stabilizes significantly more spillover than anatase, but has the largest band gap of the three supports. The anatase and alumina band gaps differ by ∼3 eV, yet neither stabilize significant amounts of spillover. A relatively small bulk band gap (i.e., a reducible support) therefore appears to be a necessary, but not sufficient, condition to stabilize spillover. The details of support surface chemistry must therefore dominate spillover stabilization.

**Figure 5:**
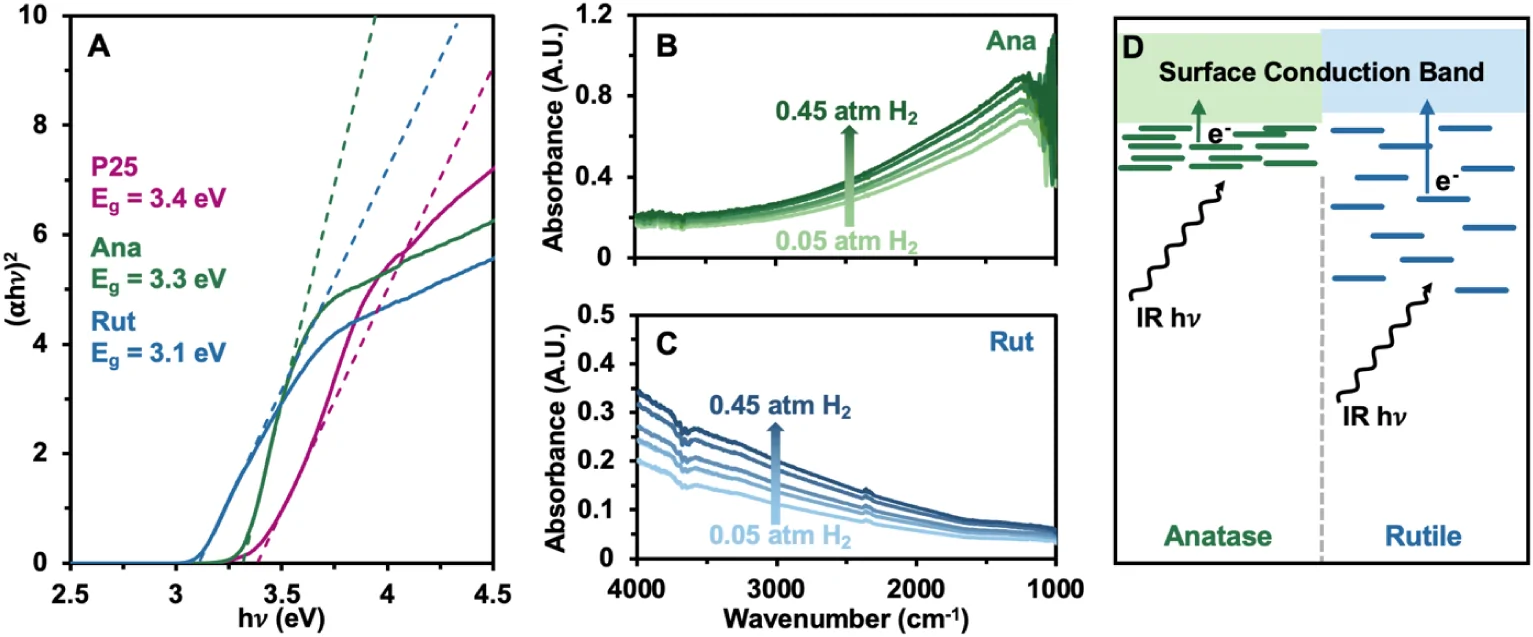
(A) Tauc plots calculated from DR UV–vis spectra of P25 (purple), anatase (green), and rutile (blue) diluted with KBr. E_g_ is calculated from the x-intercept of the linear portion. In situ FTIR difference spectra collected during H_2_ adsorption at 160 °C on (B) 1% Au/ana and (C) 1% Au/rut at 0.05, 0.10. 0.15, 0.30, and 0.45 atm H_2_. Each color shows three overlapping spectra equilibrated at the given pressure. (D) Schematic energy diagram showing the BBA origins, where H* electrons are injected into surface conduction band edge (sCBE) states and subsequently excited to the oxide surface conduction band (sCB) by irradiation with IR frequencies. The IR spectra suggest the rutile sCBE states are lower in energy than the anatase sCBE states, but this scheme is not precisely to scale.

#### Surface Electronic States

Figure 5B–C shows difference FTIR spectra of 1% Au catalysts collected under several H_2_ pressures. H_2_ adsorption results in the observed upshift in spectral baseline, which we term broad background absorbance (BBA).^16–18^ The FTIR and volumetric adsorption data are all self-consistent (calibration plots available in the SI), and nearly identical adsorption isotherms are measured in both IR and volumetric adsorption measurements.^40^

The BBA spectra of Au/ana (Figure 5B) and Au/rut (Figure 5C) differ dramatically. The Au/ana BBA signal increases at lower wavenumbers, while Au/rut shows the opposite trend—BBA intensity increases with the incident radiation energy. The spectral changes provide information on the interaction between H* and the titania surface, providing a sensitive probe for surface electronics.

Based on insights from Yates,^41–43^ Morris,^44–46^ and Frederick,^47,48^ upon adsorption, it appears the H* electrons are injected into TiO_2_ surface conduction band edge (sCBE) states. Infrared radiation excites these electrons from the sCBE into the conduction band. Because the electrons are able to access a range of sCBE states, the spectral shifts appear as change in the background absorbance across a wide range of wavelengths.

The spectral changes indicate subtle differences in the energetic wells of the surface sCBE states. The anatase surface states undergo a low energy IR transition, suggesting they are close in energy to the conduction band. Conversely, the rutile spectra indicate deeper, more stable surface sCBE states (Figure 5D). Thus, the higher H* capacity of rutile correlates with what appear to be somewhat more stable surface CBE states.

#### Surface Acid–Base Chemistry

The surface hydroxyl data (Table 2) provide insight into proton stabilization. First, there is no correlation between the total surface hydroxyl density (measured via aqueous titration) and the H* capacity. The acid–base character of anatase and rutile, however, differ substantially.^49^ While the hydroxyl K_a_ and K_b_ values are similar on all the TiO_2_ phases, rutile has the highest ratio of basic:acidic hydroxyls. This gives rutile an essentially neutral isoelectric point (IEP), which improves H* proton affinity relative to anatase.

**Table 2:** Titania Characterization Data

	Au/Rut	Au/P25	Au/Ana
**Band Gap (eV)^a^**	3.108 ± 0.002	3.385 ± 0.007	3.3163 ± 0.0005
**IEP (pH)^b^**	7.15 ± 0.06	5.24 ± 0.03	5.5 ± 0.4
**Acidic MOH (OH/nm** ^**2**^)	1.16 ± 0.03	1.79 ± 0.03	7.26 ± 0.09
**Basic MOH (OH/nm** ^**2**^)	1.34 ± 0.02	0.62 ± 0.04	1.80 ± 0.09
**Total MOH (OH/nm** ^**2**^)	3.0 ± 0.7	3.6 ± 0.3	9 ± 2
**Surface Area (m** ^**2**^ **/g)**	34 ± 1	49 ± 2	13 ± 1

a TiO_2_ Band gap calculated from DR UV-Vis.

b Isoelectric point (IEP), acidic MOH densities, and basic MOH densities determined using aqueous acid-base titrations.^49^ Surface OH densities are determined from TGA data; surface areas are from BET surface area analysis.^18,49^ All uncertainties are standard deviations from at least 3 samples.

Thermogravimetric analysis (TGA) data coupled with in situ IR spectroscopy (Figure 6), provide information on the relative distribution of surface hydroxyls and strongly adsorbed water (SAW).^18^ The SAW is defined as water that desorbs above 120 °C, attributed to adsorption on Lewis acid sites. FTIR spectra of anatase at 130 and 160 °C (Figure S11) show spectra with only one distinct peak at ∼3700 cm^–1^ (assigned to the terminal TiOH stretch). In contrast, rutile spectra contain two OH stretching features, including a broad peak at ∼3400 cm^–1^ (Figures 6A, S11). Rutile also has far fewer adsorbed hydroxyls (Figure 6B) and significantly more SAW (Figure 6C).

**Figure 6:**
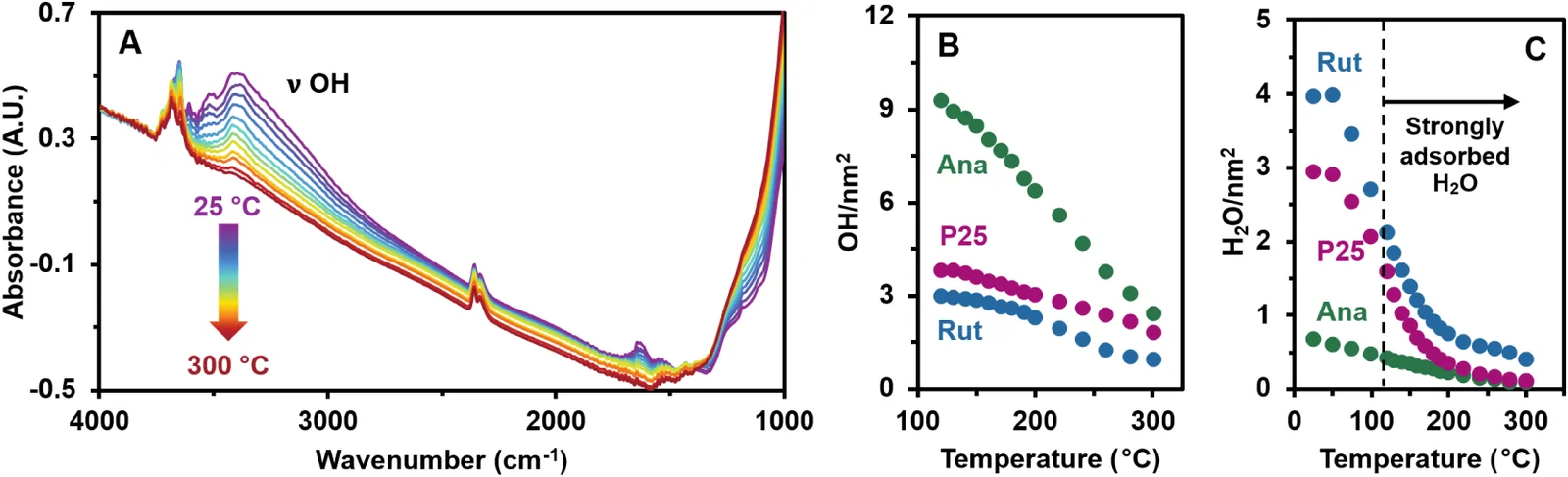
(A) FTIR spectra of rutile TiO_2_ during treatment from 25 to 300 °C. Spectra are processed with an air background and shifted on the *y*-axis so that the OH stretching regions are easier to see. (B) Hydroxyl and (C) water densities vs temperature calculated from TGA data of P25 (purple), rut (blue), and ana (green). Relative to anatase, rutile has ∼6× greater H_2_O/nm^2^ at 25 °C and 4× greater at 120 °C, indicating significantly higher surface Lewis acid site density.

The support H* capacity does not show clear trends with the water/hydroxyl speciation (Figure S12). Rutile has the highest SAW density and the lowest total hydroxyl density. However, P25 is similar to rutile in both regards, yet its H* adsorption capacity is close to anatase. While changes in hydroxyl and SAW density have important effects on H* capacity for an individual catalyst,^16–18^ these results indicate neither is a determining factor for the differences between catalysts.

The surface speciation, acid–base chemistry, and IR spectroscopy largely probe the enthalpic stability of protons and electrons that comprise H*. Anatase and rutile are generally similar, with three notable differences: (i) the relative surface concentrations of acidic and basic surface hydroxyls; (ii) the relative densities of surface hydroxyls and SAW; and (iii) the surface CBE states on rutile are somewhat more stable than on anatase. This suggests the intrinsic enthalpic stability (ΔH_ads_) for H* on anatase, rutile, and P25 are likely to be similar, with rutile perhaps showing a slightly more favorable adsorption enthalpy due to its ability to stabilize added protons and electrons. However, while surface basicity may be important, preliminary experiments show addition of NaOH to P25 titania does not improve H* capacity. This suggests the intrinsic acid–base chemistry of support hydroxyls, not general surface basicity, increases H* capacity. It is unclear if surface electronics or support acid–base chemistry is more important for spillover; both likely impact H* behavior to some degree.

The van’t Hoff studies yield an extremely favorable apparent H* adsorption entropy on rutile. This indicates the adsorption thermodynamics are dominated by the entropy of the rutile surface. While the origins of this higher surface entropy are not completely clear, it is likely tied to the larger number of readily exchangeable protons. These arise from the significantly higher amounts of SAW and the relatively balanced populations of acidic and basic surface hydroxyls. This provides a large pool of nearly thermoneutral Brønsted acid–base sites. Shifts among the proton populations of those sites provide a substantial surface entropy that should respond to changes in temperature, thus providing a larger entropic driving force for spillover on rutile. Computational studies by Pacchioni, Selloni, and coworkers comparing anatase and rutile support this finding, showing rutile has a more stable bulk structure, but less stable surface chemistry.^50–52^ Comparison of TiO_2_ surface stability also provides validation for our hypothesis regarding stability of electrons in sCBE states.

We note this analysis only applies to a specific support sample or batch, because the fine details of the support surface chemistry, such as hydroxyl Brønsted acid–base chemistry, surface area, Lewis acidity, and water content, have significant influence on spillover behavior. For example, rutile stabilizes far more spillover than anatase, which is nearly inert toward spillover. Generalizing conclusions regarding spillover-active supports or extending conclusions from one titania to another is therefore unlikely to be productive. This helps to explain why the literature is so varied on what supports are active for spillover, as details may vary widely based on the temperatures, pressures, and support batch used in individual experiments.

## Conclusions

Metal identity has little impact on the quantity or thermodynamics of hydrogen spillover, which is fundamentally a property of the catalyst support. Once H_2_ is activated/adsorbed, the entire surface (metal + support) becomes rapidly equilibrated to maintain a constant hydrogen chemical potential across all surfaces. This manifests as a single adsorption equilibrium constant at a given pressure and temperature, but different pressure dependent H coverages on the metal and support. Thus, differential adsorption on each surface changes with pressure, with the enthalpically more favorable metal adsorption dominating at low pressures regimes and spillover dominating incremental adsorption at higher pressures.

With this understanding, hydrogen adsorption can be evaluated on catalysts containing an active metal on a support capable of stabilizing spillover. This has not previously been possible. A gold catalyst can quantify the pressure and temperature dependent H* coverage on a specific support; this is subsequently used to determine M-H adsorption on more active metals (Pt, Pd, Ru, etc.). This treatment yields reasonable M-H stoichiometries (<1 H per surface metal atom).

FTIR spectroscopy and acid–base data indicate rutile more effectively stabilizes surface protons and electrons relative to anatase. The bulk electronic differences between the phases are relatively small, although the rutile surface electronic states appear to be somewhat more stabilized relative to the surface electronic states in anatase. Rutile also benefits from improved proton affinity due to the higher surface concentration of basic hydroxyl groups.

The largest difference between the supports appears to be in the temperature dependent entropic changes to the support surface itself. Van’t Hoff analyses provide evidence the rutile surface entropy is considerably higher than either anatase or P25. Considering all the data, two factors appear to dominate the higher rutile surface entropy: (i) rutile’s roughly neutral IEP, which arises from the balanced acid–base character of the support hydroxyls, and (ii) the larger amount of strongly adsorbed water. This supplies the surface with a large pool of readily exchangeable protons; this nearly thermoneutral proton exchange has inherently greater surface entropy. Incorporating surface entropy, balancing proton affinity and surface equilibria, and managing the surface electronic structure therefore provide clear avenues for tailoring supports to promote or inhibit spillover.

## Materials and Methods

### Chemicals

Argon, and O_2_ gases were UHP grade and were supplied by Linde. HAuCl_4_·3H_2_O (99.7%) was purchased from Sigma-Aldrich. NH_4_OH (29.3%) was purchased from Fisher Scientific. Urea (99.5%) was purchased from Acros Organics. Evonik P25 TiO_2_ (49 m^2^ /g surface area, 0.47 cm^3^/g pore volume) was generously supplied by Evonik. Anatase TiO_2_ was purchased from Alfa Aesar (13 m^2^/g surface area, 0.08 cm^3^/g pore volume). Rutile TiO_2_ was purchased from Thermo Fisher (34 m^2^/g surface area). Gamma Al_2_O_3_ (CATALOX HP 14/150) was purchased from Sasol (144 m^2^/g surface area, 0.9 cm^3^/g pore volume). K_2_PtCl_4_ was purchased from Sigma-Aldrich. H_2_ gas for adsorption experiments was provided by an NM Plus electrolytic H_2_ gas generator from VICI DBS. The generator was supplied with Milli-Q water (18.2 MΩ·cm at 25 °C), which was also our source of deionized (DI) water for all syntheses. Both the argon and hydrogen gases were scrubbed of H_2_O and oxygen using a moisture and oxygen scavenging trap purchased from Restek USA.

[Au­(en)_2_]­Cl_3_ was synthesized by a previously described method.^53^ Briefly, HAuCl_4_ (396 mg, 1.0 mmol) was dissolved in 10 mL DI H_2_O. Ethylene diamine (en) was then added as 2 mL of a 1.0 M solution (136 μL, 2,0 mmol), forming a dark brown precipitate. After stirring at room temperature for 1 hour, precipitate dissolved. Then 80 mL ethanol were added, and the mixture was allowed to cool in a refrigerator for ∼30 min. The yellow-brown product was then isolated by centrifuging at 8,000 rpm for 5 min and washed with 2 × 15 mL cold ethanol before drying.

### Catalyst Synthesis

All catalysts were synthesized at nominally 1 wt.% metal loading. Exact metal loading was determined with X-ray fluorescence (XRF) spectroscopy on a Malvern Panalytical Epsilon 4 Spectrometer. All catalysts were sealed in vials and placed in a refrigerator in the dark for storage. Au catalyst synthesis was based on several previously reported methods.^54–56^

#### 1% Au/P25

1 wt.% Au supported on P25 TiO_2_ was synthesized in a round-bottom flask by deposition-precipitation with urea. 0.10 g HAuCl_4_ and 0.50 g urea were dissolved in 85 mL DI H_2_O. The yellow solution was heated with stirring until the temperature reached 80 °C (10–15 min). 5.0 g of P25 TiO_2_ were then added, and the mixture was stirred and heated at 80 °C for 4 h. After 4 h, the stirring was stopped, and the mixture was cooled to room temperature. The solid with isolated by centrifugation (6,000 rpm, 25 min) and washed with 40 mL 0.1 M NH_4_OH and then 15 × 40 mL DI H_2_O. The product was then washed with more H_2_O until the filtrate reached pH ∼ 7. Upon the final wash, the filtrate was tested with AgNO_3_ to confirm that no Cl^–^ was detected. The solid was dried at room temperature overnight, then reduced at 275 °C under 5% H_2_ in N_2_ for 4 h.

#### 1% Au/Rutile

1 wt.% Au supported on rutile TiO_2_ was synthesized in a round-bottom flask by deposition-precipitation with urea. 0.040 g HAuCl_4_ and 0.20 g urea were dissolved in 35 mL DI H_2_O. The yellow solution was heated with stirring until the temperature reached 80 °C (10–15 min). 2.0 g of rutile TiO_2_ were then added, and the mixture was stirred and heated at 80 °C for 4 h. After 4 h, the stirring was stopped, and the mixture was cooled to room temperature. The solid with isolated by centrifugation (6,000 rpm, 25 min) and washed with 40 mL 0.1 M NH_4_OH and 10 × 40 mL H_2_O. Upon the final wash, the filtrate was tested with AgNO_3_ to confirm that no Cl^–^ was detected. The solid was dried at room temperature overnight, then reduced at 275 °C under 5% H_2_ in N_2_ for 4 h.

#### 1% Au/Anatase

1 wt.% Au supported on anatase TiO_2_ was synthesized in a round-bottom flask by strong electrostatic adsorption (SEA). 48 mg [Au­(en)_2_]­Cl_3_ was dissolved in 60 mL DI H_2_O (1.7 mM). Then, 2.0 g anatase TiO_2_ was dispersed in 40 mL DI H_2_O and brought to 70 °C. The [Au­(en)_2_]­Cl_3_ solution was added dropwise and the mixture was stirred at 70 °C for 1 h. After cooling to room temperature, 30 mL of 1 M NH_4_OH were added, and the mixture was stirred for additional hour. The solid was washed ∼15 times with 40 mL DI H_2_O each by centrifuge (4000 rpm, 30 min) until pH reached 7 and no Cl^–^ remained (confirmed by testing with AgNO_3_).

#### 1%Au/γ-Al_2_O_3_

1 wt.% Au supported on γ-Al_2_O_3_ was synthesized in a round-bottom flask by deposition-precipitation with urea. 0.060 g HAuCl_4_ and 0.30 g urea were dissolved in 50 mL DI H_2_O. The yellow solution was heated with stirring until the temperature reached 80 °C (10–15 min). 3.0 g of γ-Al_2_O_3_ were then added, and the mixture was stirred and heated at 80 °C for 4 h. After 4 h, the stirring was stopped, and the mixture was cooled to room temperature. The solid with isolated by centrifugation (6,000 rpm, 25 min) and washed with 11 × 40 mL H_2_O and 40 mL 0.1 M NH_4_OH. The product was then washed with more H_2_O until the filtrate reached pH ∼ 7. Upon the final wash, the filtrate was tested with AgNO_3_ to confirm that no Cl^–^ was detected. The solid was dried at room temperature overnight, then reduced at 275 °C under 5% H_2_ in N_2_ for 4 h.

#### 1% Pt/Rutile

1 wt.% Pt supported on rutile TiO_2_ was synthesized in a round-bottom flask. 0.050 g K_2_PtCl_4_ was dissolved in 15 mL DI H_2_O. The solution was heated with stirring until the temperature reached 60 °C (10 min). 2.0 g of rutile TiO_2_ were then added, and the mixture was stirred and heated at 60 °C for 1 h. After 1 h, the stirring was stopped, and the mixture heated to 90 °C until all of the water evaporated (overnight). The product was then washed with 1.0 M NH_4_OH once and DI H_2_O at least 4 times to remove remaining Cl. The light gray product was oxidized for 3 h at 350 °C under 20% O_2_, then reduced at 300 °C under 5% H_2_ in N_2_ for 4 h.

Additional catalysts were synthesized by the same method with appropriate quantities of K_2_PtCl_4_ to produce 0.1, 0.3, and 0.5 wt.% Pt/rutile.

#### 1% Pt/γ-Al_2_O_3_

1 wt.% Pt supported on γ-Al_2_O_3_ was synthesized by SEA based on literature procedures.^57^ Briefly, 9.2 × 10^–4^ M of dilute K_2_PtCl_6_ in water adjusted to a pH of 4 using HCl was added to γ-Al_2_O_3_ (Sasol CATALOX HP 14/150). The solution was stirred at ambient conditions for 1 h before being washed with 3 times with water, 5 times 4:1 acetone:water, and 1 time with acetone via centrifugation. The product was then washed with 1.0 M NH_4_OH once and DI H_2_O at least 4 times to remove remaining Cl. The resulting solid was dried under vacuum overnight then calcined (air, 350 °C, 5 h, 5 °C/min, 50 mL/min) and reduced (5% H_2_, 350 °C, 4 h, 5 °C/min, 50 mL/min).

### Volumetric Chemisorption (Micromeritics 3Flex)

#### H_2_ Adsorption Procedure

Hydrogen adsorption experiments were performed on a Micromeritics 3Flex instrument. ∼500 mg of previously pretreated catalyst (i.e., Au precursor was already reduced) was evacuated at 300 °C for 10 min then oxidized under flowing O_2_ for 30 min. After evacuating for 10 min, the samples were reduced under flowing H_2_ at 160 °C for 30 min. The sample was then evacuated, purged with helium for 3 h, and cooled to the analysis temperature under an active vacuum. H_2_ adsorption isotherms were measured at least twice at 130 and 160 °C for all samples. The sample was evacuated to less than 10 μm Hg for 1 h at the adsorption temperature between isotherms.

### In Situ FTIR Spectroscopy

#### HTC and Flow Setup

FTIR spectra were collected on a Bruker Invenio-R FTIR spectrometer in a heated (20–500 °C) transmission flow cell (Harrick Instruments). Gas flows were controlled using mass flow controllers (Bronkhorst) which were calibrated for each gas using a soap and bubble flow meter. H_2_O and oxygen in the feed gases were minimized with moisture and oxygen scavenging traps (Restek USA). For the H_2_ adsorption measurements, ∼20 mg of catalyst sample was pressed (1 ton of pressure for 24 s) into 100 × 100 mesh stainless steel gauze (Unique Wire Weaving Co.) with a hydraulic press. The resulting sample pellet was 13 mm in diameter and was mounted in the flow cell.

#### H_2_ Adsorption Procedure

The sample pellet was pretreated in situ to remove surface adsorbates (primarily carbonates) by flowing 40 mL/min of argon over the catalyst at 300 °C for 30 min. The feed was then switched to 20% O_2_/Ar (40 mL/min total flow) for 30 min, then to 40 mL/min argon for 30 min. The sample pellet was cooled to ambient temperature under 40 mL/min argon; the sample was left under slowly flowing Ar overnight. Catalysts treated weekly with this pretreatment showed no sign of diminished H_2_ adsorption activity over 3–4 months. Pretreated samples were heated to 160 °C to eliminate weakly adsorbed water. The catalyst was rereduced in 40% H_2_/Ar at 160 °C for 1 hour, then purged with 40 mL/min Ar flow for 2 h to remove all adsorbed H_2_ (low-temperature reduction).

A detailed description of the materials and methods used is available in the SI.

## Supplementary Material



## Notes

Methods and materials; STEM images and nanoparticle size analysis; material characterization; calibration of H_2_ adsorption with FTIR and volumetric chemisorption; equilibrium constant calculations; quantification of surface-adsorbed water and hydroxyls (PDF)
