# Supplementary material for: A Quantitative Examination of Catalyst Component Impacts on Hydrogen Adsorption and Spillover
Source: J Am Chem Soc. 2026 Aug 4;148(32):34550–9. doi: 10.1021/jacs.6c08946 (PMC13495768; doi:10.1021/jacs.6c08946)
Supplement: Supplementary file 1 [file ja6c08946_si_001.pdf]

Supporting Information for

A quantitative examination of catalyst component impacts on hydrogen adsorption and spillover

Audrey M. Battiste[1], Tae Yong Yun[2], Kelle D. Hart[1], Rebekah J. Snellings[1], Mohammad Hamidizirasefi[2], Angela Pathickal Abraham[1], Bert D. Chandler[,1,2]*

[1]Department of Chemistry, The Pennsylvania State University, University Park, Pennsylvania 16802, United States

[2]Department of Chemical Engineering, The Pennsylvania State University, University Park, Pennsylvania 16802, United States

* To whom correspondence should be addressed:
bert.chandler@psu.edu; phone (814) 863-6229

Table of Contents

SI.1. Experimental Section *3*

SI.1.1.Chemicals *3*

SI.1.2. Catalyst Synthesis *3*

SI.1.3. Characterization *5*

SI.1.3.1. Powder XRD Patterns *5*

SI.1.3.2. Band Gap and BET Surface Area *5*

SI.1.3.3. STEM Images and NP Size Analysis *6*

SI.1.3.4. Acid-Base Titration *8*

SI.1.3.5. XRF Elemental Analysis *8*

SI.1.3.6 Thermogravimetric Analysis *9*

SI.1.3.7 Temperature Programmed Reduction *10*

SI.2. Isotherm Measurements *11*

SI.2.1. Volumetric H_2 Chemisorption *11*

SI.2.2. In Situ FTIR Spectroscopy *13*

SI.2.2.1. HTC and Flow Setup *13*

SI.2.2.2. H_2 Adsorption Procedure *13*

SI.2.2.3. BBA Calibration *16*

SI.3. Thermodynamic Calculations *17*

SI.3.1. K_θ Calculations *17*

SI.3.2. Van't Hoff Analysis *19*

SI.4. References *19*

SI.1. Experimental

SI.1.1. Chemicals

Argon, and O_2 gases were UHP grade and were supplied by Linde. $HAuCl_4•3H_2O$ (99.7%) was purchased from Sigma-Aldrich. NH_4OH (29.3%) was purchased from Fisher Scientific. Urea (99.5%) was purchased from Acros Organics. Evonik P25 TiO_2 (49 m^2 /g surface area, 0.47 cm^3/g pore volume) was generously supplied by Evonik. Anatase TiO_2 was purchased from Alfa Aesar (13 m^2/g surface area, 0.08 cm^3/g pore volume). Rutile TiO_2 was purchased from Thermo Fisher (34 m^2/g surface area). Gamma Al_2O_3 (CATALOX HP 14/150) was purchased from Sasol (144 m^2/g surface area, 0.9 cm^3/g pore volume). K_2PtCl_4 was purchased from Sigma Aldrich. H_2 gas for adsorption experiments was provided by an NM Plus electrolytic H_2 gas generator from VICI DBS. The generator was supplied with Milli-Q water (18.2 MΩ•cm at 25 °C), which was also our source of de-ionized (DI) water for all syntheses. Both the argon and hydrogen gases were scrubbed of H_2O and oxygen using a moisture and oxygen scavenging trap purchased from Restek USA.

$[Au(en)_2]Cl_3$ was synthesized by a previously described method.[1] Briefly, $HAuCl_4$ (396 mg, 1.0 mmol) was dissolved in 10 mL DI H_2O. Ethylene diamine (en) was then added as 2 mL of a 1.0M solution (136μL, 2,0 mmol), forming a dark brown precipitate. After stirring at room temperature for one hour, precipitate dissolved. Then 80 mL ethanol were added, and the mixture was allowed to cool in a refrigerator for ~30 minutes. The yellow-brown product was then isolated by centrifuging at 8,000 rpm for 5 minutes and washed with 2 x 15 mL cold ethanol before drying.

SI.1.2. Catalyst Synthesis

Catalyst syntheses shown below are calculated at nominally 1 wt.% metal loadings. Additional metal loadings were synthesized by changing the metal precursor quantity accordingly. Loadings were determined with x-ray fluorescence (XRF) spectroscopy. All catalysts sealed in vials and stored in a refrigerator in the dark.

1% Au/P25

1 wt.% Au supported on P25 TiO_2 was synthesized in a round bottom flask by deposition-precipitation with urea. 0.10 g $HAuCl_4$ and 0.50 g urea were dissolved in 85 mL DI H_2O. The yellow solution was heated with stirring until the temperature reached 80˚C (10-15 minutes). 5.0 g of P25 TiO_2 were then added, and the mixture was stirred and heated at 80˚C for 4 hours. After 4 hours, the stirring was stopped, and the mixture was cooled to room temperature. The solid with isolated by centrifugation (6,000 rpm, 25 minutes) and washed with 40 mL 0.1 M NH_4OH and then 15 × 40 mL DI H_2O. The product was then washed with more H_2O until the filtrate reached pH ~7. Upon the final wash, the filtrate was tested with $AgNO_3$ to confirm that no Cl^- was detected. The solid was dried at room temperature overnight, then reduced at 275 ˚C under 5% H_2 in N_2 for 4 hours.

1% Au/rutile

1 wt.% Au supported on rutile TiO_2 was synthesized in a round bottom flask by deposition-precipitation with urea. 0.040 g $HAuCl_4$ and 0.20 g urea were dissolved in 35 mL DI H_2O. The yellow solution was heated with stirring until the temperature reached 80˚C (10-15 minutes). 2.0

g of rutile TiO_2 were then added, and the mixture was stirred and heated at 80˚C for 4 hours. After 4 hours, the stirring was stopped, and the mixture was cooled to room temperature. The solid with isolated by centrifugation (6,000 rpm, 25 minutes) and washed with 40 mL 0.1 M NH_4OH and 10 × 40 mL H_2O. Upon the final wash, the filtrate was tested with $AgNO_3$ to confirm that no Cl^- was detected. The solid was dried at room temperature overnight, then reduced at 275 ˚C under 5% H_2 in N_2 for 4 hours.

1% Au/anatase

1 wt.% Au supported on anatase TiO_2 was synthesized in a round bottom flask by strong electrostatic adsorption (SEA). 48 mg $[Au(en)_2]Cl_3$ was dissolved in 60 mL DI H_2O (1.7 mM). Then, 2.0 g anatase TiO_2 was dispersed in 40 mL DI H_2O and brought to 70 °C. The $[Au(en)_2]Cl_3$ solution was added dropwise and the mixture was stirred at 70 °C for 1 hour. After cooling to room temperature, 30 mL of 1 M NH_4OH were added, and the mixture was stirred for additional hour. The solid was washed ~15 times with 40 mL DI H_2O each by centrifuge (4000 rpm, 30 minutes) until pH reached 7 and no Cl^- remained (confirmed by testing with $AgNO_3$).

1% Au/Al_2O_3

1 wt.% Au supported on P25 TiO_2 was synthesized in a round bottom flask by deposition-precipitation with urea. 0.060 g $HAuCl_4$ and 0.30 g urea were dissolved in 50 mL DI H_2O. The yellow solution was heated with stirring until the temperature reached 80˚C (10-15 minutes). 3.0 g of Al_2O_3 were then added, and the mixture was stirred and heated at 80˚C for 4 hours. After 4 hours, the stirring was stopped, and the mixture was cooled to room temperature. The solid with isolated by centrifugation (6,000 rpm, 25 minutes) and washed with 11 × 40 mL H_2O and 40 mL 0.1 M NH_4OH. The product was then washed with more H_2O until the filtrate reached pH ~7. Upon the final wash, the filtrate was tested with $AgNO_3$ to confirm that no Cl^- was detected. The solid was dried at room temperature overnight, then reduced at 275 ˚C under 5% H_2 in N_2 for 4 hours.

1% Pt/rutile

1 wt.% Pt supported on rutile TiO_2 was synthesized in a round bottom flask. 0.050 g K_2PtCl_4 was dissolved in 15 mL DI H_2O. The solution was heated with stirring until the temperature reached 60˚C (10 minutes). 2.0 g of rutile TiO_2 were then added, and the mixture was stirred and heated at 60˚C for 1 hour. After 1 hour, the stirring was stopped, and the mixture heated to 90 °C until all of the water evaporated (overnight). The product was then washed with 1.0 M NH_4OH once and DI H_2O at least 4 times to remove remaining Cl. The light gray product was oxidized for 3 hours at 350 °C under 20% O_2, then reduced at 300 ˚C under 5% H_2 in N_2 for 4 hours.

Additional catalysts were synthesized by the same method with appropriate quantities of K_2PtCl_4 to produce 0.1, 0.3, and 0.5 wt.% Pt/rutile.

1% Pt/Al_2O_3

1 wt.% Pt supported on Al_2O_3 was synthesized by SEA based on literature procedures.[2] Briefly, 9.2 x 10^{-4} M of dilute K_2PtCl_6 in water adjusted to a pH of 4 using HCl was added to Al_2O_3 (Sasol CATALOX HP 14/150). The solution was stirred at ambient conditions for 1 h before being washed with 3 times with water, 5 times 4:1 acetone:water, and 1 time with acetone via centrifugation. The product was then washed with 1.0 M NH_4OH once and DI H_2O at least 4 times

to remove remaining Cl. The resulting solid was dried under vacuum overnight then calcined (air, 350 °C, 5 h, 5 °C/min, 50 mL/min) and reduced (5% H_2, 350 °C, 4 h, 5 °C/min, 50 mL/min).

SI.1.3 Characterization

SI.1.3.1 Powder XRD patterns

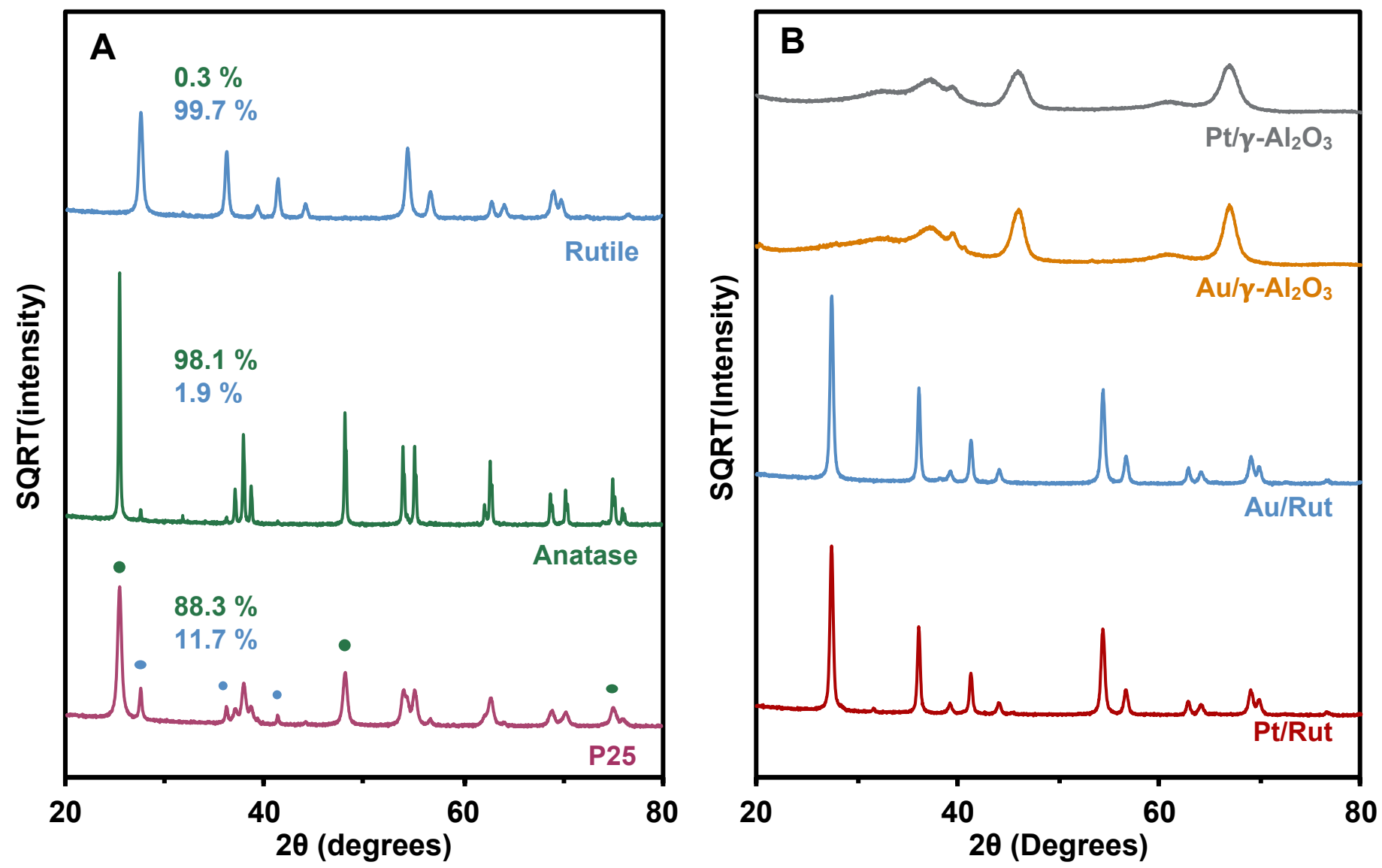

Figure S1: (A) Powder XRD patterns of rutile (blue), anatase (green), and P25 (purple). Phase compositions (calculated from Rietveld refinement) are listed by percent of rutile (blue) and anatase (green) for all three TiO_2 samples. Key anatase and rutile peaks in the P25 pattern are highlighted with a dot of the corresponding color. **(B)** Powder XRD patterns of the four types of catalysts used in this study: Pt/rutile TiO_2 (red), Au/rutile TiO_2 (blue), Au/γ-Al_2O_3 (gold), and Pt/γ-Al_2O_3 (gray). All patterns have been shifted to arbitrary y-axis values, but relative intensities have not been altered.

SI.1.3.2. Band Gap and BET Surface Area

Table S1: oxide band gaps calculated from Tauc plots of diffuse-reflectance UV-vis spectra. Surface areas are calculated from BET specific surface area analysis. Uncertainties are the standard deviations for data from 3 distinct samples of each material.

Oxide	Band gap (eV)	Surface Area (m^2/g)
P25 TiO_2	3.39 ± 0.01	49 ± 2
Rutile TiO_2	3.110 ± 0.002	34 ± 1
Anatase TiO_2	3.317 ± 0.001	13 ± 1
Al_2O_3	5.690 ± 0.004	144 ± 6

SI.1.3.3. STEM images and NP size analysis

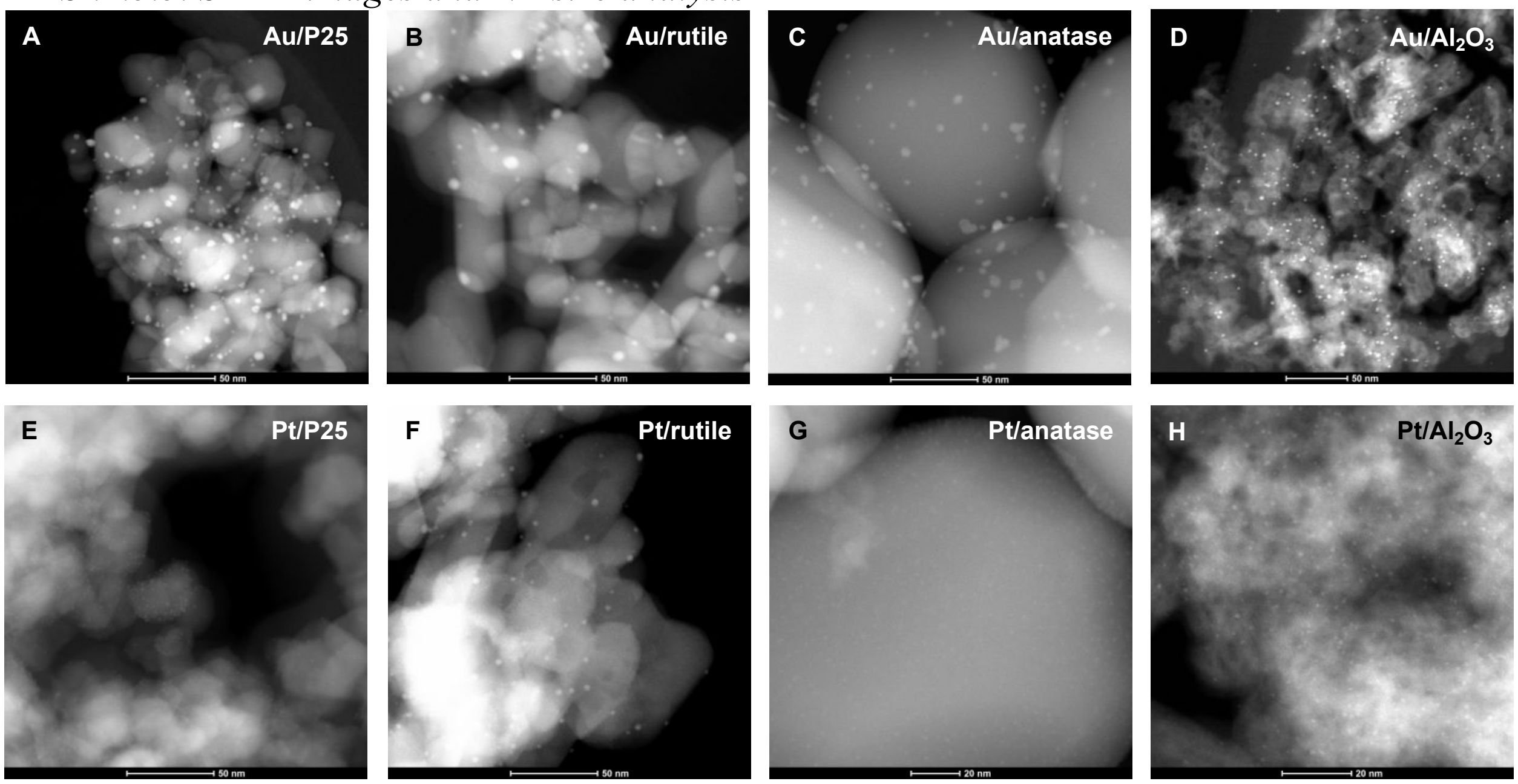

Figure S2: STEM images of 8 catalysts.

Gold particle sizes were determined by imaging with scanning/transmission electron microscopy (STEM) using a Talos F200C microscope at an accelerating voltage of 200 kV. To prepare each sample, a small quantity of catalyst was crushed in an agate mortar and pestle and dispersed in isopropanol. A Cu TEM grid (Ted Pella, Inc.) was dipped into this suspension and allowed to dry before analysis. The average Au or Pt nanoparticle diameter was determined using ImageJ from the measurement of >300 particles (**Figure S2**).

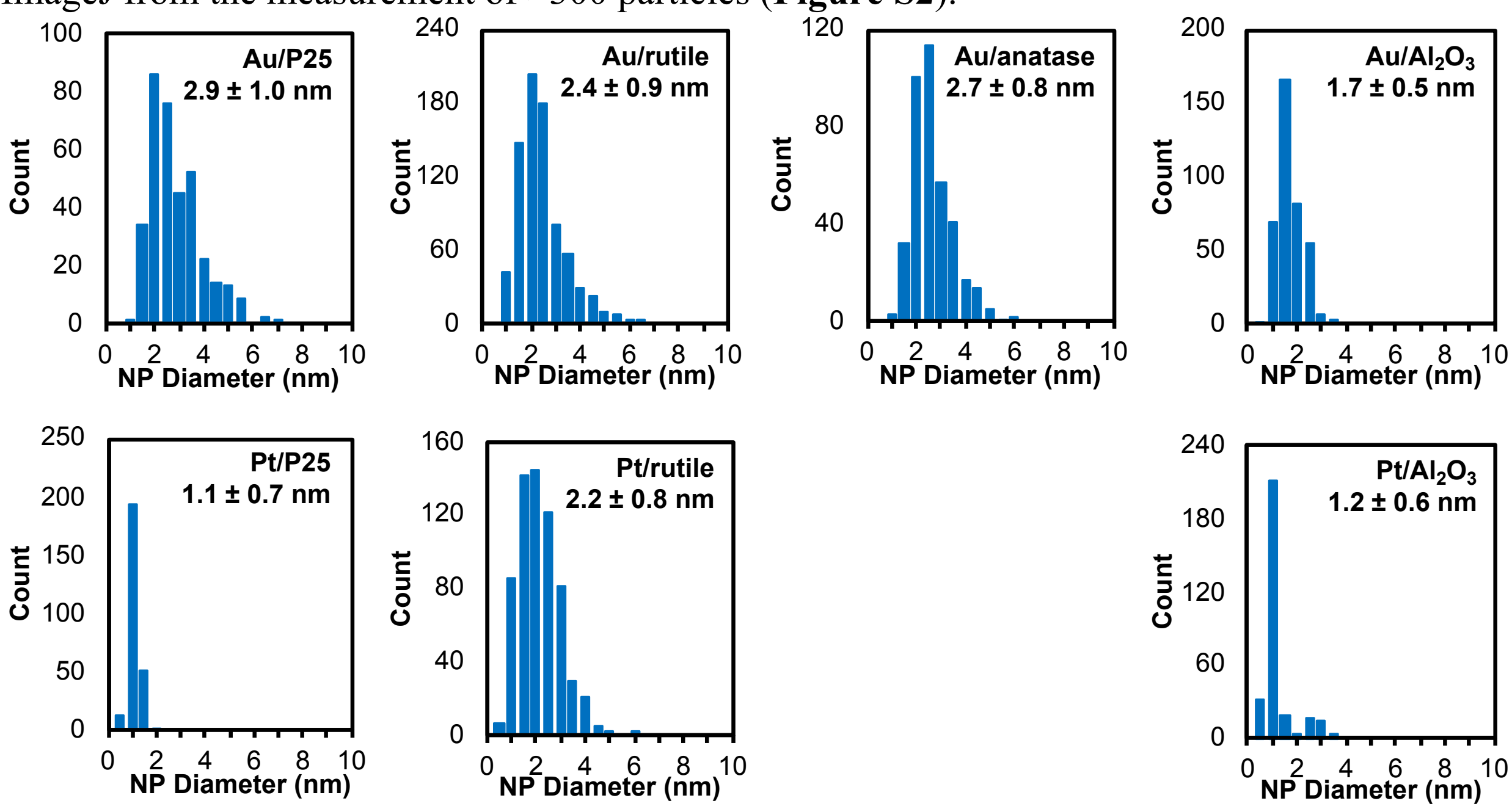

Figure S3: Histograms of metal nanoparticle diameters measured in ImageJ from STEM images of >300 nanoparticles on each catalyst. Average particle size is listed in each plot area.

Uncertainties are standard deviations of all nanoparticles measured on the respective sample. Pt/ana particles are small enough to be monodisperse, so a histogram is not shown; Pt dispersion is considered to be 100% for this catalyst.

Au and Pt Perimeter and Surface Site Analysis:

The fraction of Au or Pt atoms on the surface (%S) or perimeter (%P) of the nanoparticles was determined using previously reported methods modeling Au and Pt particles as cuboctahedra.[3] Perimeter site density (M_{per}), surface site density (M_{surf}), %P, and %S were calculated using **Equations S1-S4**.

$$\%P = \frac{24 m_M}{5 D_p^2 r_M \rho_M \sqrt{2}} \times 100 \quad \text{(S1)}$$

$$\%S = \frac{12 m_M (3+\sqrt{3})}{5 \pi D_p r_M^2 \rho_M \sqrt{2}} \times 100 \quad \text{(S2)}$$

$$M_{per}\left(\frac{\mu mol}{g\ cat.}\right) = \frac{\%M \times \%Per \times 10^6}{M_w(M)} \quad \text{(S3)}$$

$$M_{sur}\left(\frac{\mu mol}{g\ cat.}\right) = \frac{\%M \times \%Sur \times 10^6}{M_w(M)} \quad \text{(S4)}$$

Where:

- m_M is the mass of a single metal atom (3.27×10^{-25} kg/Au atom, 3.24×10^{-25} kg/Pt atom)
- d_p is the circumsphere diameter of a cuboctahedral particle (from TEM)
- r_M is the atomic radius of Au (1.66×10^{-10} m/atom) or Pt (1.39×10^{-10} m/atom)
- ρ_M is the bulk density of Au (19300 kg/m^3) or Pt (21450 kg/m^3)

Calculating %P using mean nanoparticle diameter from a wide distribution of particle sizes leads to significant error due to the non-linear relationship between %P and D_p. Instead, %P was calculated for the entire particle size histogram, considering more than 300 nanoparticles. For perimeter site density determination, bin sizes of 0.25 nm were used, calculating the %P based on the center of each bin. These values were then multiplied by their respective bin count and averaged to estimate the weighted average %P of the samples shown in **Table S2**. The same process was followed to calculate weighted average %S of each sample. Further derivations and justifications pertaining to this analysis can be found in the SI of our previous reports.[3,4] While the average dNP is listed (and commonly used) for Au samples, the distribution of NP diameters (**Figure S3**) has a significant impact on %S calculations. For example, while Au/P25 and Au/ana have similar dNP averages, Au/P25 has significantly more NPs with $d \leq 2$ nm. Because %S is proportional to 1/diameter^2, these small NPs contribute more to the number of surface cites than larger NPs.

Table S2: Average nanoparticle size, surface metal (M_{surf}) distribution, and perimeter metal (M_{per}) distribution. Uncertainties are calculated from standard deviations.

Catalyst	Average d_{NP} (nm)	%S	%P	M_{surf} (µmol/g)	M_{per} (µmol/g)
1% Au/P25	2.9 ± 1.0	43	5	22	2.5
1% Au/rutile	2.4 ± 0.9	28	6	14	3.0
1% Au/anatase	2.7 ± 0.8	16	6	16	3.2
1% Au/Al_2O_3	1.7 ± 0.5	49	15	20	5.9

2% Pt/P25	1.1 ± 0.7	65	36	56	31
1% Pt/rutile	2.2 ± 0.8	66	10	41	6.2
1% Pt/anatase	<1	~100	~100	15	15
1% Pt/Al_2O_3	1.2 ± 0.6	75	38	31	16

SI.1.3.4. Acid-base titrations

Acid-base titrations were performed as described extensively in our previous publication.[5]

SI.1.3.5. XRF Elemental Analysis

Table S4: XRF spectroscopy was used determine metal loadings on M/MOx catalysts. Collected on a Malvern Panalytical Epsilon 4 Spectrometer.

Catalyst (Nominal)	M wt. %
1% Pt/Al2O3	0.815
1% Au/Al2O3	1.196
1% Pt/rut	1.204
1% Au/rut	1.14
2% Pt/P25	1.683
1% Au/P25	0.925
1% Pt/ana	0.302
1% Au/ana	1.271

SI.1.3.6 Thermogravimetric Analysis (TGA)

TGA experiments were performed on a TA Instruments Discovery TGA 550-Water™ analysis instrument. A stepwise decarbonation and heating process under 99.999% nitrogen (40 mL/min, Linde) was used to quantify water and hydroxyl loss. The sample was purged with N_2 at 25 °C for 60 minutes, then heated to 300 °C (heating rate = 10 °C/min) and held at 300 °C for 1 hour to remove surface carbonates. Then, the sample was cooled to room temperature (RT) and held for 8 hours to re-hydroxylate the surface. After 8 hours, the temperature increased to 50 °C at a heating rate of 5 °C/min and held at 50 °C for 30 minutes. Next, the temperature increased to 75 °C at a heating rate of 5 °C/min and held for 30 minutes. This heating-holding protocol was then repeated for the following temperatures: 100, 120, 130, 140, 150, 160, 170, 180, 190, 200, 220, 240, 260, 280, and 300 °C. After the hold at 300 °C, the temperature was ramped to 600 °C at 10 °C/min and held for 30 minutes.

Dehydration & dehydroxylation During FTIR Experiments:

Identical decarbonation and heating process was used to correlate FTIR spectral changes to sample mass loss in TGA. After decarbonation at 300 ℃ for 1 hour, the temperature was cooled to RT and held for 8 hours to re-hydroxylate the surface. Then, a background spectrum was collected under 40 mL/min of Ar at RT, and the temperature increased to 50 ℃. Then a sample

scan was collected. After 10 minutes another sample scan was collected under 40mL/min Ar and processed using the same background. The sample scans were compared; if the scans showed no measurable changes, then the sample was heated to the desired dehydration & dihydroxylation temperature under 40mL/min Ar. Next, this heating-holding protocol with FTIR scan was repeated for the following temperatures: 75, 100, 120, 130, 140, 150, 160,170, 180, 190, 200, 220, 240, 260, 280, and 300 °C.

Calibration of TGA & FTIR:

The ν_{OH} (3580~3760 cm^{-1}) and δ_{HOH} (1580~1660 cm^{-1}) band losses from FTIR spectra (such as those seen in **Figure 7A** in the main text) were integrated and plotted as a function of temperature (see **Figure S4**).

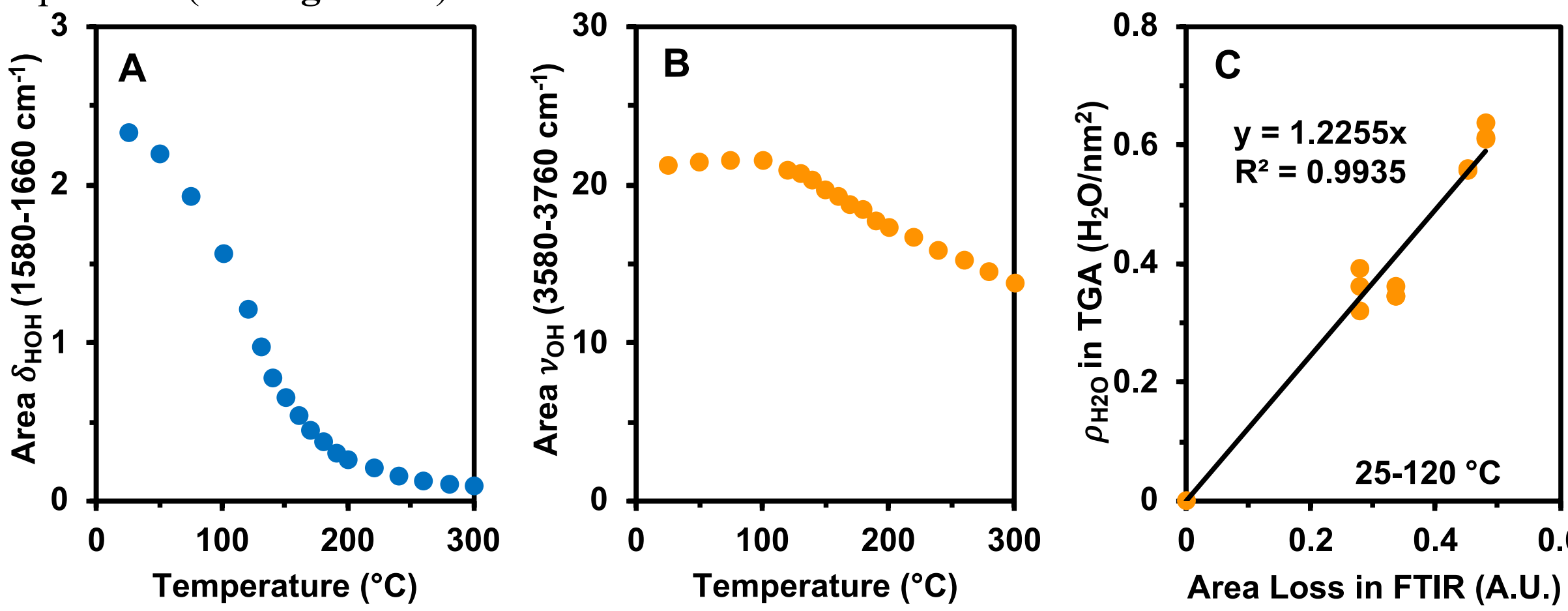

Figure S4: Example calibration data for P25 TiO_2. **(A)** Integrated area of ν_{OH} peak in FTIR spectra. **(B)** Integrated area of δ_{HOH} peak in FTIR spectra. **(C)** Calibration of δ_{HOH} area to TGA mass loss.

Here, since the hydroxyl regime remained unchanged up to 120 ˚C (**Figure S3B**), the measured mass loss from 25 to 120 ˚C in the TGA corresponds to the desorption of adsorbed water. Thus, the losses of ρ_{H_2O} in this temperature range were calculated based on TGA mass losses. Plotting losses of ρ_{H_2O} against area losses in FTIR at same temperature (See **Figure S4C**) reveals strong linear correlation, allowing us to calibrate and quantify the integration of δ_{HOH} (1580~1660 cm^{-1}) in terms of the quantity of adsorbed water at other temperatures. The converted ρ_{H_2O} versus temperature data is presented in **Figure 7C** of the main text. To further distinguish between the desorption of strongly adsorbed water and dehydroxylation in the TGA beyond 120 ˚C, the calculated ρ_{H_2O} are subtracted from the total TGA mass losses. From this, ρ_{TiOH} at different temperatures are determined using following **Equation S14** and plotted in **Figure 7B** of the main text.

$$\#\frac{OH}{nm^2} = \frac{\left(\#\frac{OH}{nm^2}\right)_{T2} * SSA * m_{T2} + \left[\frac{(m_{T1}-m_{T2})}{\frac{MW_{H2O}}{NA}} * 2\right]}{SSA * m_{T1}}$$

(S5)

Where m_{T1} and m_{T2} are the sample mass at the corresponding temperature T_1 and T_2 respectively, MW_{H2O} is the molecular weight of water, N_A is Avogadro's constant, and SSA is specific surface area.

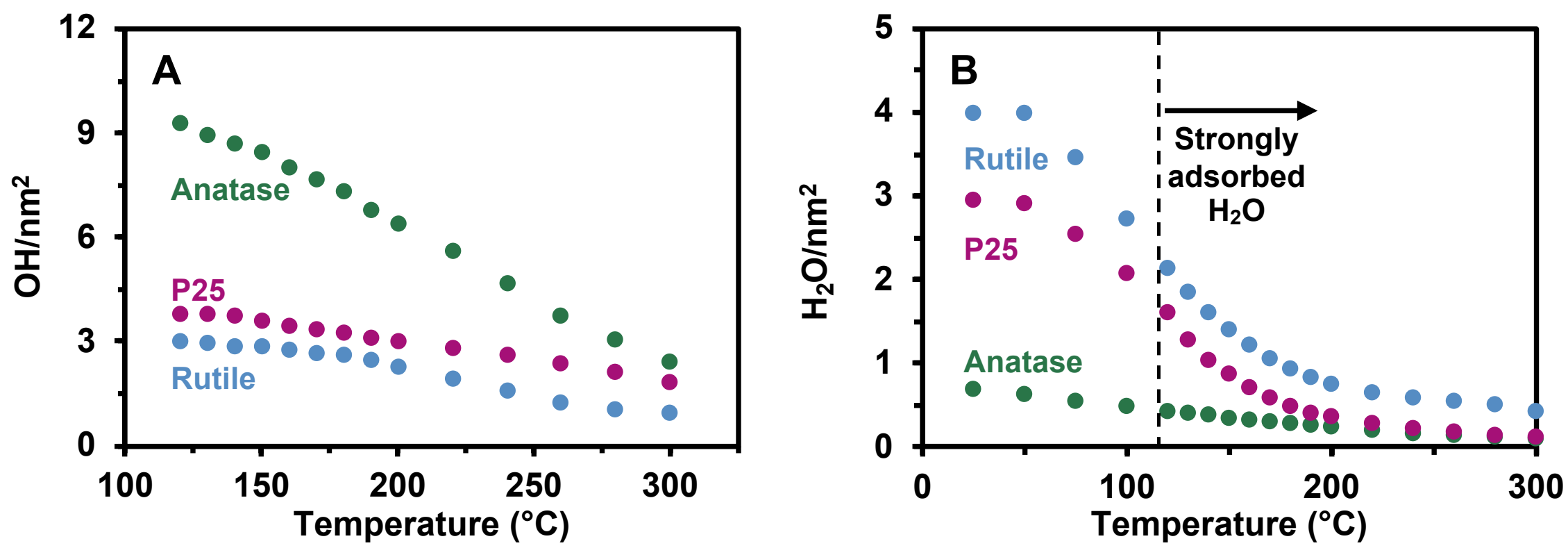

Figure S5: (A) Hydroxyl density of anatase (green) and rutile (blue) from TGA data, calculated as described above. **(B)** Water density calculated as described above.

SI.1.3.7 Temperature Programmed Reduction (TPR)

TPR experiments were performed on TiO_2 supported catalysts. This analysis shows no significant support reduction up to 600 °C. Samples were calcined in situ at 300 °C and cooled to room temperature under inert atmosphere before measurement. Ramp rate was 10 °C/min for all measurements.

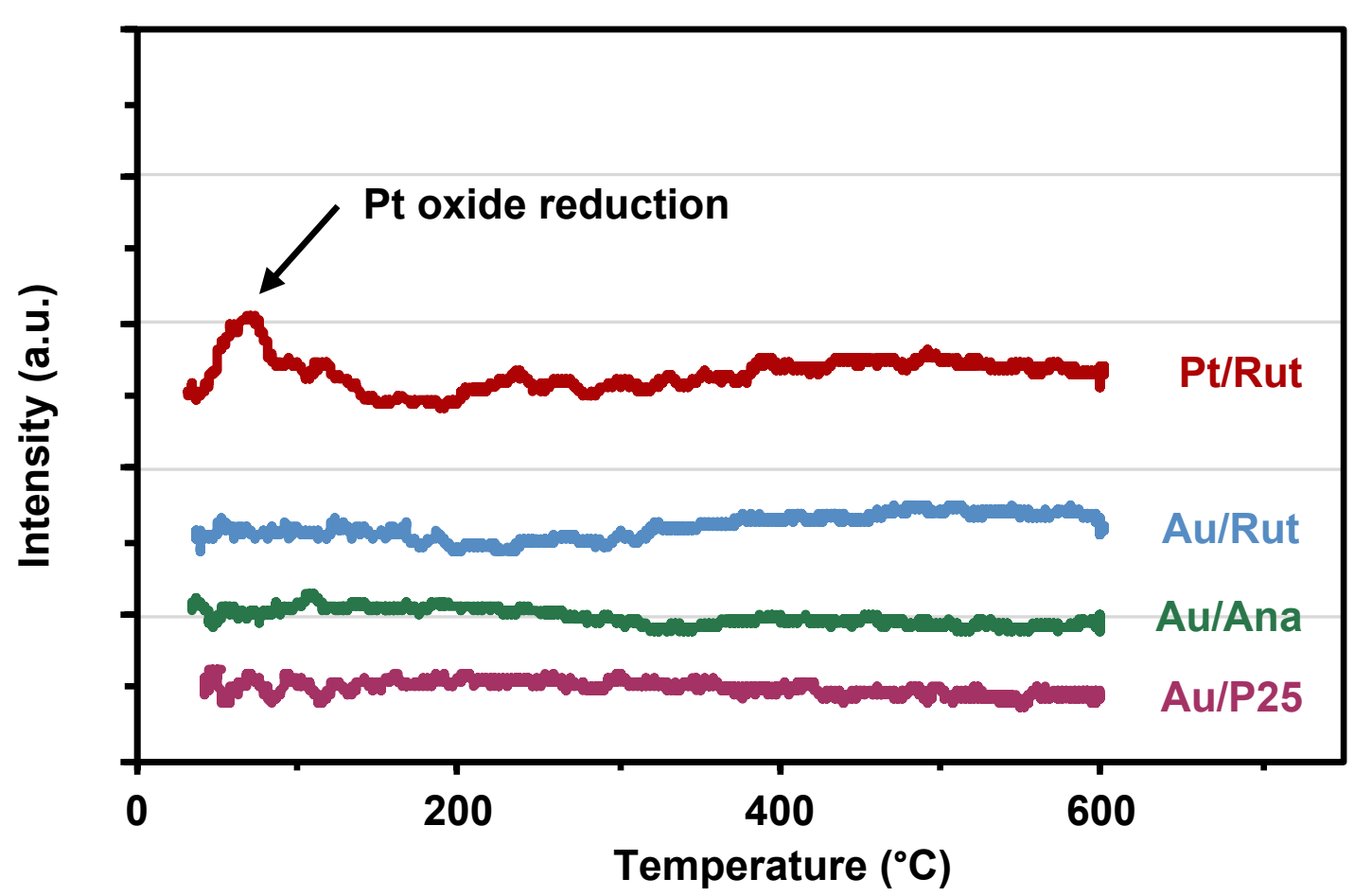

Figure S6: TPR measurements of Pt/rutile (red), Au/rutile (blue), Au/anatase (green), and Au/P25 (purple) catalysts, as described above.

SI.2 Isotherm Measurements

SI.2.1. Volumetric H_2 Chemisorption (Micromeritics 3Flex)

Hydrogen adsorption experiments were performed on a Micromeritics 3Flex instrument. ~500 mg of previously pretreated catalyst (i.e., Au precursor was already reduced) was evacuated at 300 °C for 10 minutes then oxidized under flowing O_2 for 30 minutes. After evacuating for 10 minutes, the samples were reduced under flowing H_2 at 160 °C for 30 minutes. The sample was then evacuated, purged with helium for 3 hours, and cooled to the analysis temperature under an active vacuum. H_2 adsorption isotherms were measured at least twice at 130 and 160 °C for all samples. The sample was evacuated to less than 10 μm Hg for 1 hour at the adsorption temperature between isotherms.

Hydrogen adsorption is measured in two sequences per isotherm. First, H_2 is dosed at the designated pressures and initial adsorption quantities are recorded. This adsorption isotherm is the "total" adsorption on a material. After a brief evacuation time, H_2 is added again to measure a second isotherm. Any hydrogen left on the surface after the brief evacuation is considered "strongly adsorbed", because the adsorption is not readily reversible. Subtracting the second isotherm measurement from the first results in a strong H_2 adsorption isotherm (**Figure S7 E&F**). We refer to H adsorption as primarily reversible in this work, indicating the first and second isotherms show approximately the same adsorption values.

P25 is a high surface area mixture of 88% anatase and 12% rutile, so we considered the possibility that grain boundaries might impact spillover. **Figure 3C** in the main text also compares the H_2 adsorption on P25 to a calculated isotherm (Calc-P25) determined by summing the normalized adsorption on Au/rut and Au/ana at each pressure. The experimental and calculated isotherms are identical, indicating spillover is impacted only by the phases present, not presence of grain boundaries or interactions between the phases. Thus, the composition of P25 only appears to impact spillover to the extent that it is tied to synthetic methodologies capable of generating higher surface area supports.

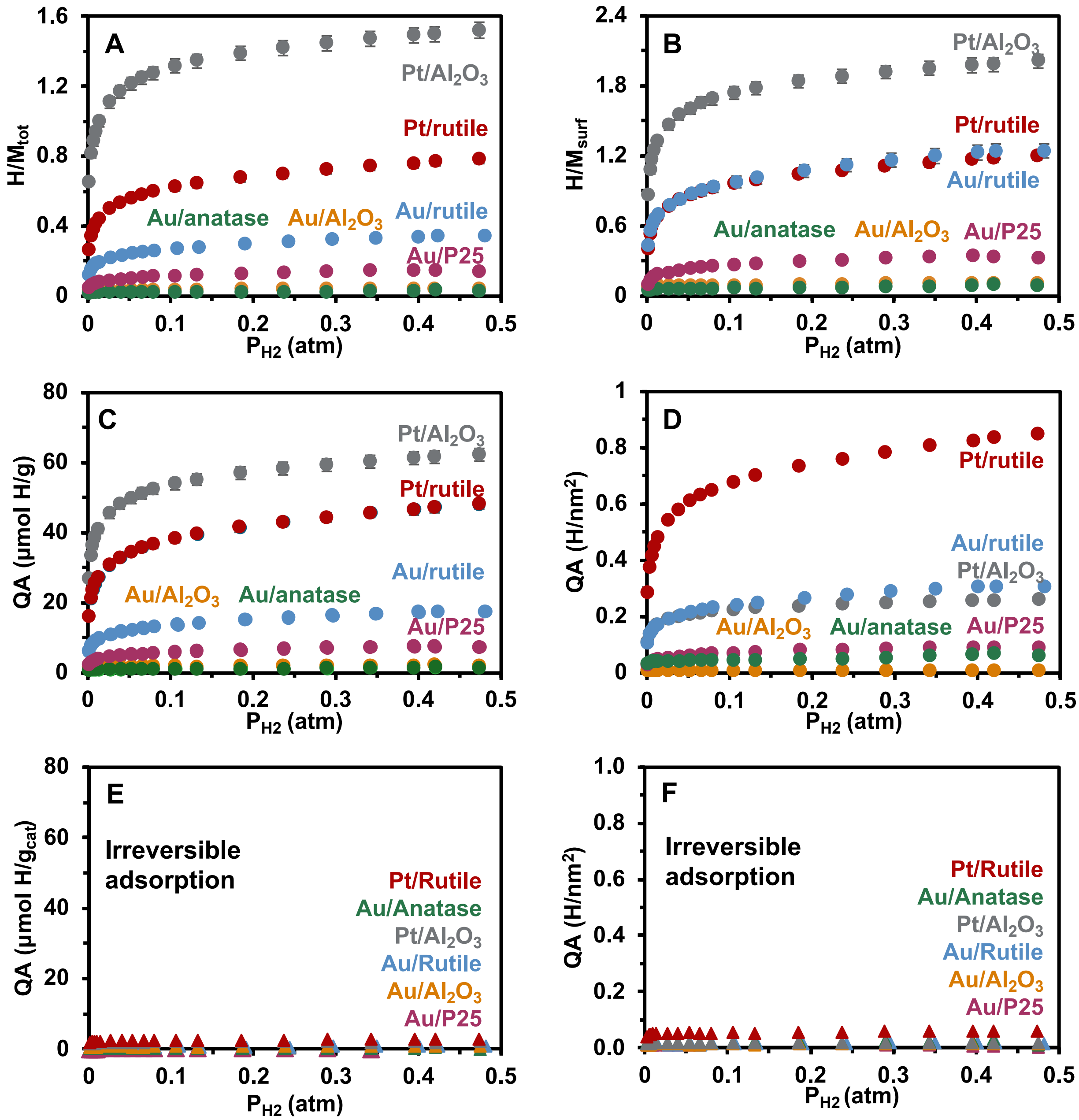

Figure S7: (A)-(D) Total H_2 adsorption isotherms on 1% M/MO$_x$ catalysts at 160 °C normalized to **(A)** total metal atoms, **(B)** surface metal atoms, **(C)** total catalyst mass, and **(D)** support surface area. Strong (irreversible) H_2 adsorption on 1% M/MO$_x$ catalysts is shown normalized to **(E)** total catalyst mass and **(F)** support surface area.

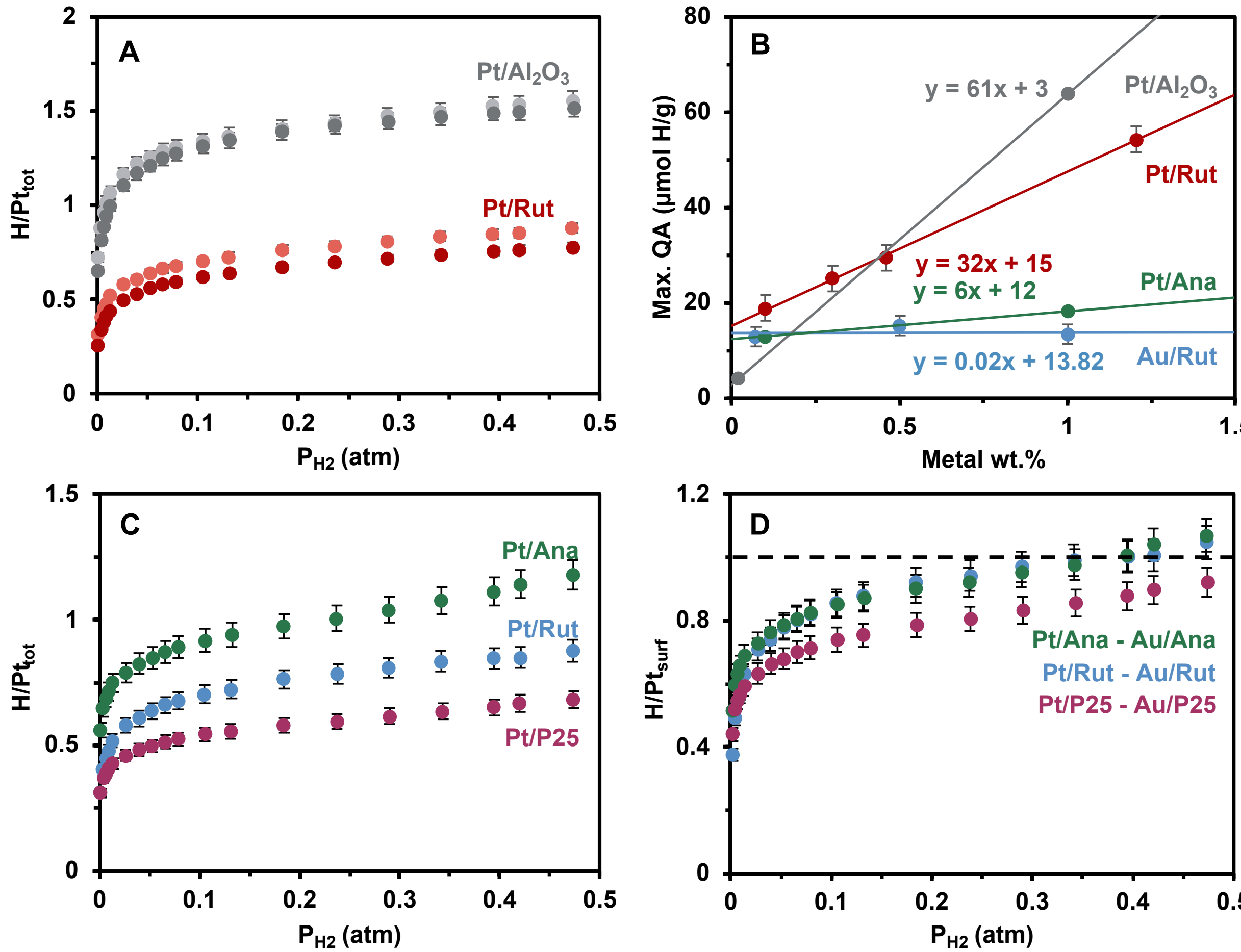

Figure S8: (A) H_2 adsorption isotherms normalized to total Pt at 160 °C (dark colors) and 130 °C (light colors) **(B)** Maximum H adsorption (P_{H2} = 0.5 atm) vs metal loading on Pt/Al_2O_3 (gray), Pt/rut (red), Pt/ana (green), and Au/rut (blue). **(C)** H_2 adsorption on Pt/TiO_2 catalysts at 130 °C normalized to total Pt atoms. **(D)** H_2 adsorption on Au/TiO_2 (spillover quantity on each support) subtracted from Pt/TiO_2 at 130 °C normalized to number of surface Pt atoms. This subtraction effectively shows adsorption quantity on only the Pt surface.

Extended Au surfaces do not activate H_2,[6–8] yet Au/Al_2O_3 is an active hydrogenation catalyst.[7,9] **Figure 1B** shows small, but measurable H_2 adsorption on Au/Al_2O_3, consistent with adsorption at a fraction of the MSI sites (details below). The Au/Al_2O_3 results confirm there is little to no spillover on alumina; we therefore attribute the Pt/Al_2O_3 data (**Figure 1B**) to adsorption on the metal.[14] The greater than unity H/Pt_{surf} ratio **(Figure S8A)** has been observed previously and attributed to a combination of (i) multiple H atoms adsorbing on surface Pt sites, and (ii) proton transfer from the metal to the MSI hydroxyls.[6,8,10]

Figures S8C-D show H adsorption on Pt/TiO_2, where **Figure S8C** is total H adsorption per Pt atom and **Figure S8D** has adsorption on the support (H adsorption on Au/ana-, rut-, or P25-TiO_2) subtracted and is normalized to Pt surface atoms (Pt_{surf}). Adsorption on the three catalysts are similar once contributions from the support are removed and Pt dispersion is accounted for, indicating H adsorption on Pt is the same regardless on TiO_2 phase, and further supporting the conclusion that spillover H behavior is independent of metal identity.

SI.2.2 In Situ FTIR Spectroscopy

SI.2.2.1. HTC and flow setup

FTIR spectra were collected on a Bruker Invenio-R FTIR spectrometer in a heated (20-500 °C) transmission flow cell (Harrick Instruments). Gas flows were controlled using mass flow controllers (Bronkhorst) which were calibrated for each gas using a soap and bubble flow meter. H2O and oxygen in the feed gases were minimized with moisture and oxygen scavenging traps (Restek USA). For the H_2 adsorption measurements, ~20mg of catalyst sample was pressed (1 ton of pressure for 24s) into 100 x 100 mesh stainless steel gauze (Unique Wire Weaving Co.) with a hydraulic press. The resulting sample pellet was 13mm in diameter and was mounted in the flow cell.

SI.2.2.2. H_2 adsorption procedure

The sample pellet was pretreated to remove surface adsorbates (primarily carbonates) by flowing 40 mL/min of argon over the catalyst at 300 °C for 30 minutes. The feed was then switched to 20% O_2/Ar (40 mL/min total flow) for 30 minutes, then to 40 mL/min argon for 30 minutes. The sample pellet was cooled to ambient temperature under 40 mL/min argon; the sample was left under slowly flowing Ar overnight. Catalysts treated weekly with this pretreatment showed no sign of diminished H_2 adsorption activity over 3-4 months. Complete removal of surface species during this treatment is evidenced below in **Figure S8**, as well as referenced in previous publications.[4,11] Pretreated samples were heated to 160 °C to eliminate weakly adsorbed water. The catalyst was re-reduced in 40% H_2/Ar at 160 °C for one hour, then purged with 40 mL/min Ar flow for 2 hours to remove all adsorbed H_2 (low-temperature reduction).

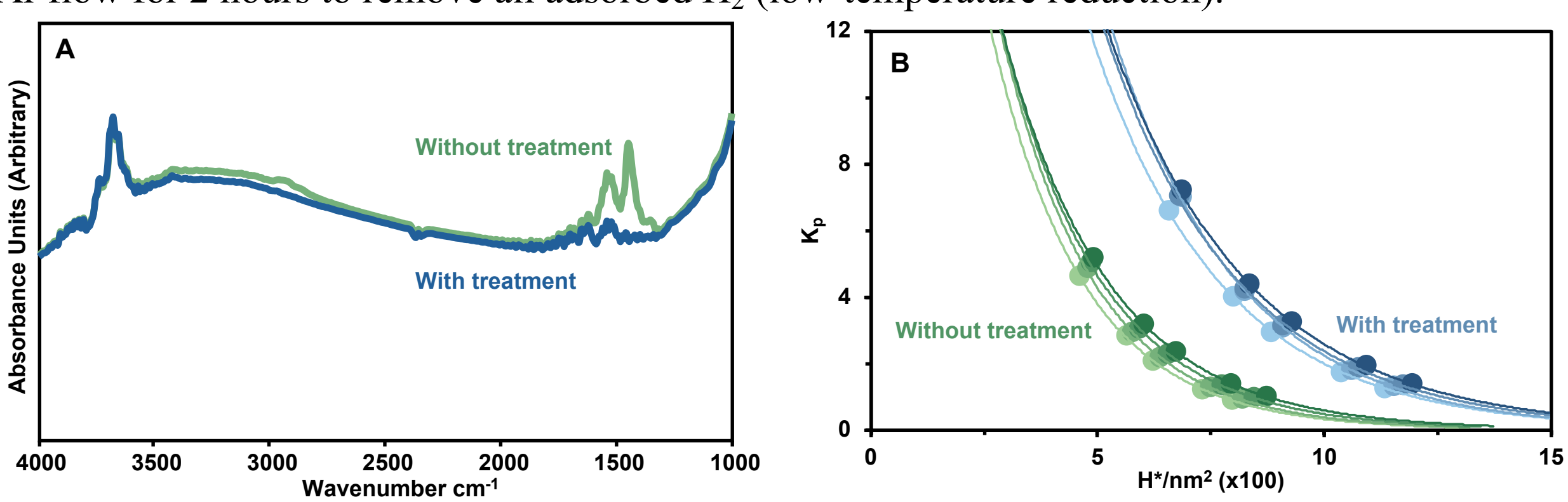

Figure S9: **(A)** FTIR spectra collected before (light blue) and after (dark blue) the pretreatment described above on a Au/P25 catalyst. Spectra are shifted for easiest comparison of peaks. **(B)** Equilibrium constants vs H* coverage with (blue) and without (green) pretreatment calculated from in situ FTIR H_2 adsorption isotherms. Isotherms were collected at four temperatures from 130 (lightest) to 160 (darkest) °C.

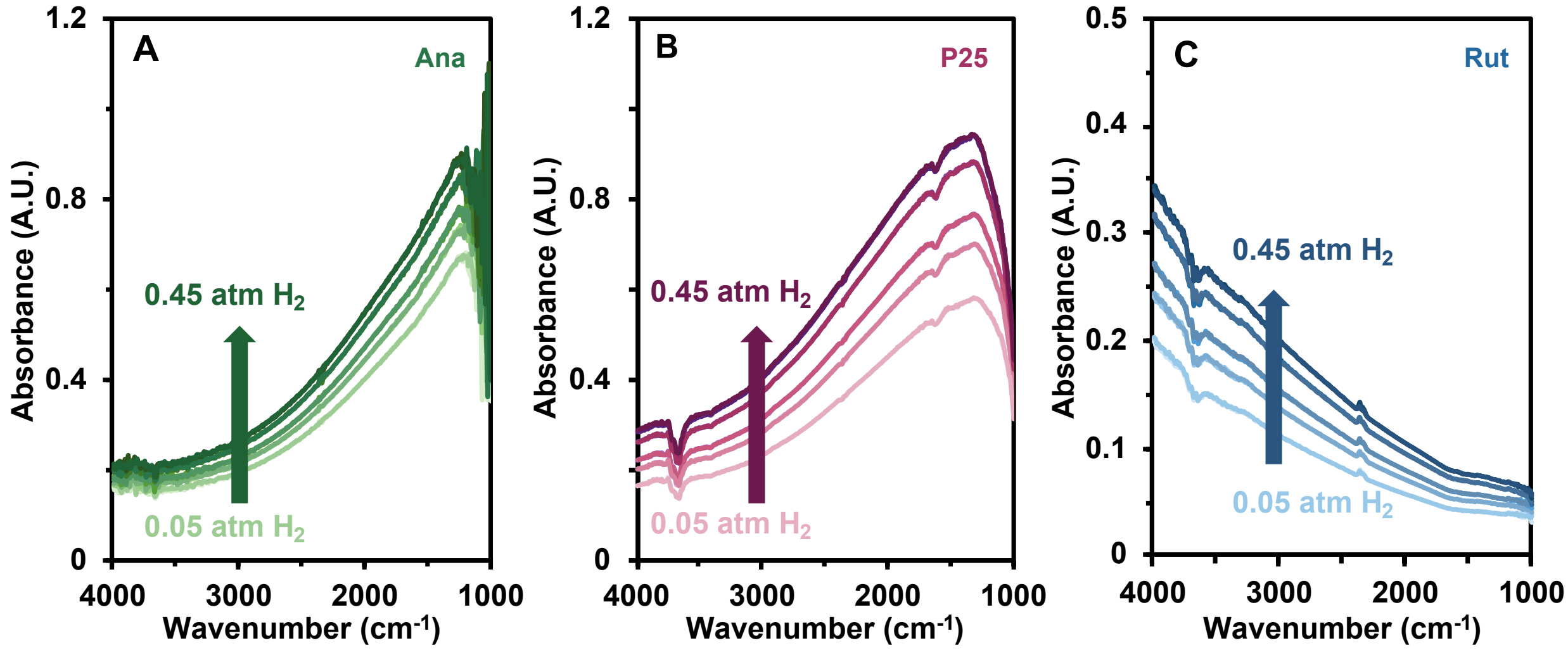

Figure S10: In situ FTIR difference spectra collected during H_2 adsorption at 160 °C on **(A)** 1% Au/ana, **(B)** 1% Au/P25, and **(C)** 1% Au/rut at 0.05, 0.10. 0.15, 0.30, and 0.45 atm H_2. Each color shows three overlapping spectra equilibrated at the given pressure. Integration of a designated area of the curve was integrated in order to calculate adsorption as described below.

In situ FTIR spectra return rapidly to the original baseline upon removal of H_2 from the gas feed, supporting the complete reversibility of H adsorption previously noted above in the volumetric chemisorption measurements.[12,13]

Spectra processed with air background:

FTIR spectra collected at 160 °C after pretreatment and before adsorbing H_2 were re-processed using a background scan taken under dry air to compare H_2O and H-bonding character on anatase and rutile (**Figure S11**).

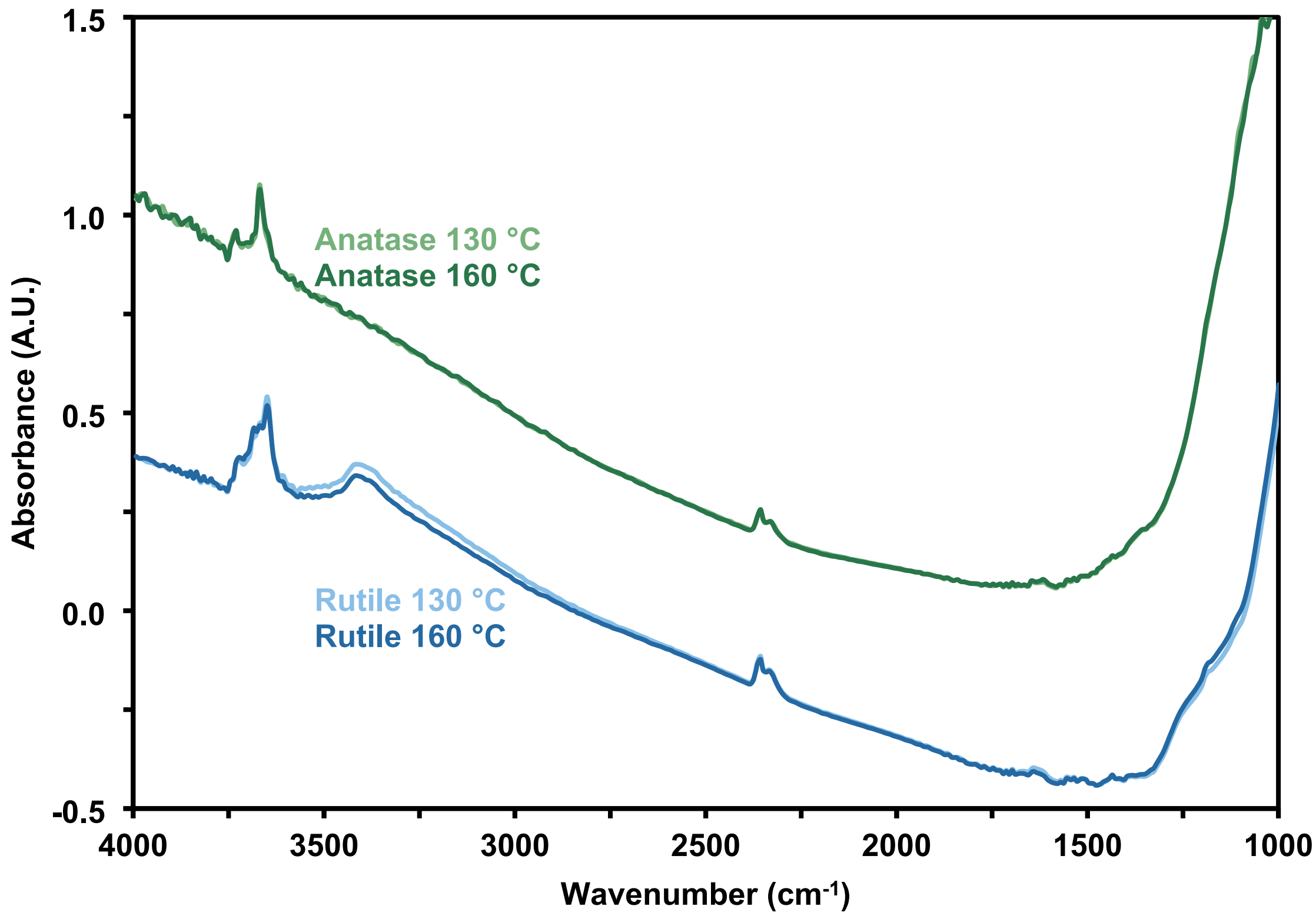

Figure S11: FTIR spectra of rutile (blue) and anatase (green) TiO_2 at 130 and 160 °C processed with an air background and baseline corrected to show changes in the H bonding and OH-stretching region.

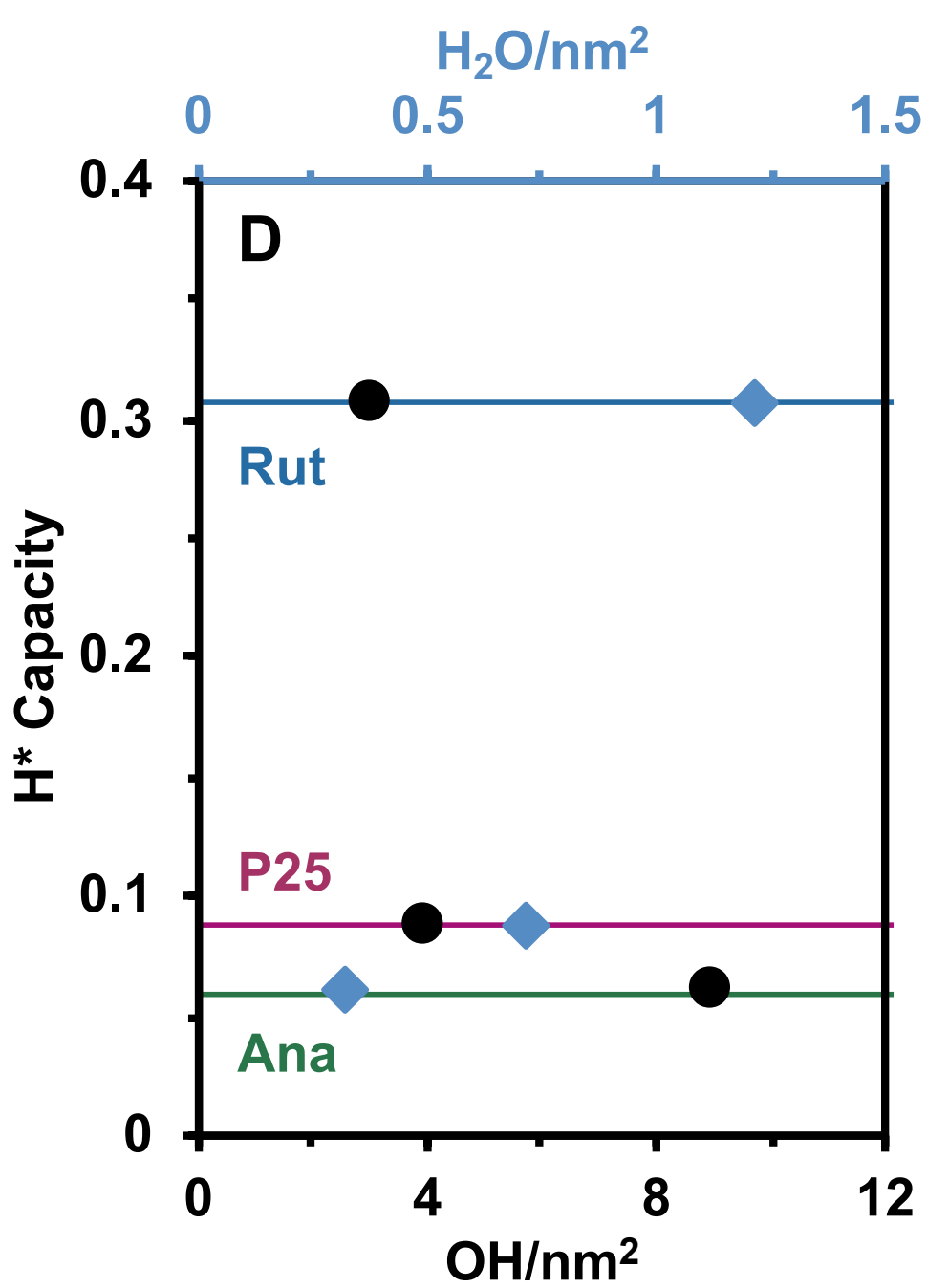

Figure S12: H* adsorption capacity at 0.45 atm H_2 and 160 °C versus surface hydroxyl density (bottom axis, black dots) and surface water density (top axis, blue diamonds) on 1% Au/TiO_2 catalysts. No clear trends emerge in comparing H* to the surface speciation.

SI.2.2.3. BBA Calibration

A simple system of equations is used to calibrate the BBA signal with the volumetric chemisorption data. An example calculation using the equations fit to the 130 and 160 °C isotherms shown in **Figure S10** is shown below. The BBA data (red) and QA data (green) are first fit to the following natural logarithm functions:

$$y = 14.7\ln(x) + 86.7 \quad \text{(S6)}$$

$$y = 1.28\ln(x) + 8.55 \quad \text{(S7)}$$

Equation S6 is then solved with respect to x.

$$e^{\frac{y-86.7}{14.7}} \quad \text{(S8)}$$

Equation S8 is then substituted into **Equation S7**.

$$y = 1.28\ln\left(e^{\frac{y-86.7}{14.7}}\right) + 8.55 \quad \text{(S9)}$$

The 6 experimental BBA values collected at 120 °C are then substituted into the red exponential equation (for y) in **Equation S9**. The resulting values are deemed "calculated QA values". Next, **Equation S7** is solved with respect to x.

$$e^{\frac{y-8.55}{1.28}} = x \quad \text{(S10)}$$

Equation S8 is then substituted into **Equation S6**.

$$y = 14.7\ln\left(e^{\frac{y-8.55}{1.28}}\right) + 86.7 \quad \text{(S11)}$$

The 6 experimental QA values collected at 130 and 160 °C are then substituted into the green exponential equation (for y) in **Equation S11**. The resulting values are deemed "calculated BBA values".

The calculated BBA and calculated QA values are the predicted values based on the trends in the experimental BBA and QA data. The BBA calibration factor is found by plotting the calculated QA values vs. the experimental BBA values and the calculated BBA values vs. the experimental QA values for the 130 and 160 °C adsorption data (**Figure S13**). The calibration factor is the resulting slope between the data sets, as shown in **Figure S13**. The integrated BBA area is multiplied by the calibration factor to convert the BBA area directly into adsorbed hydrogen (H) per gram of catalyst.

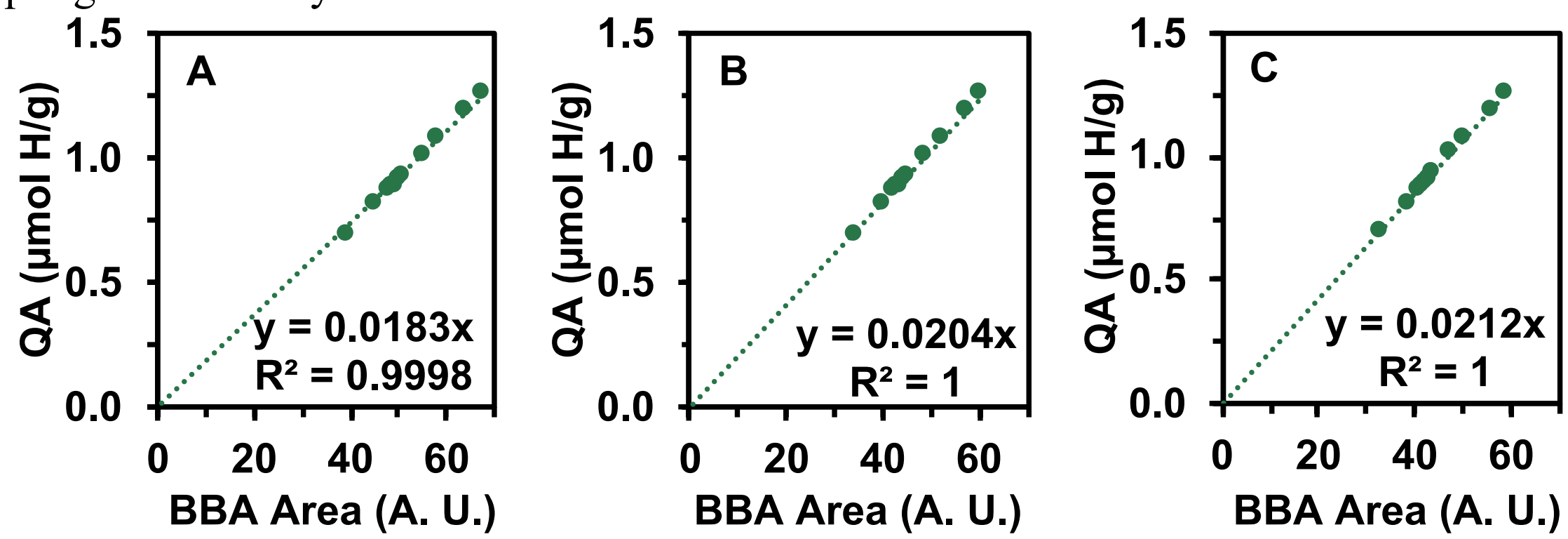

Figure S13: BBA-chemisorption calibration plots for 3 samples of 1% Au/anatase at 160 °C where the slope of each line is the calibration factor.

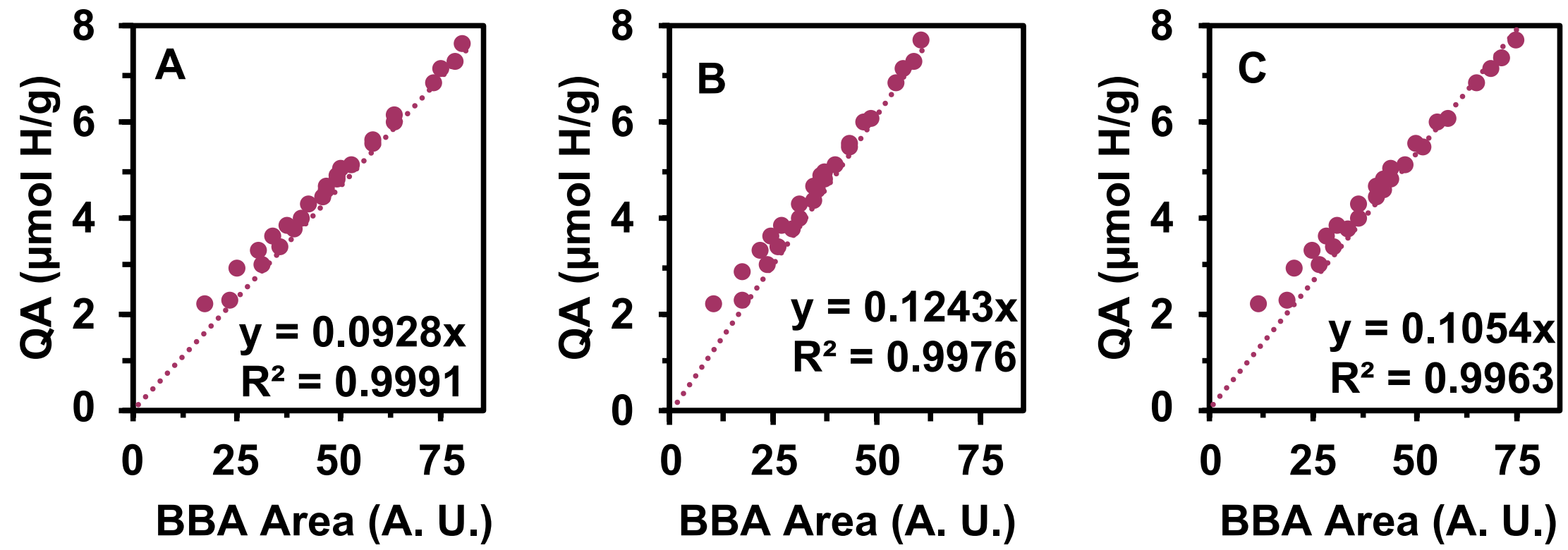

Figure S14: BBA-chemisorption calibration plots for 3 samples of 1% Au/P25 at 130 and 160 °C where the slope of each line is the calibration factor.

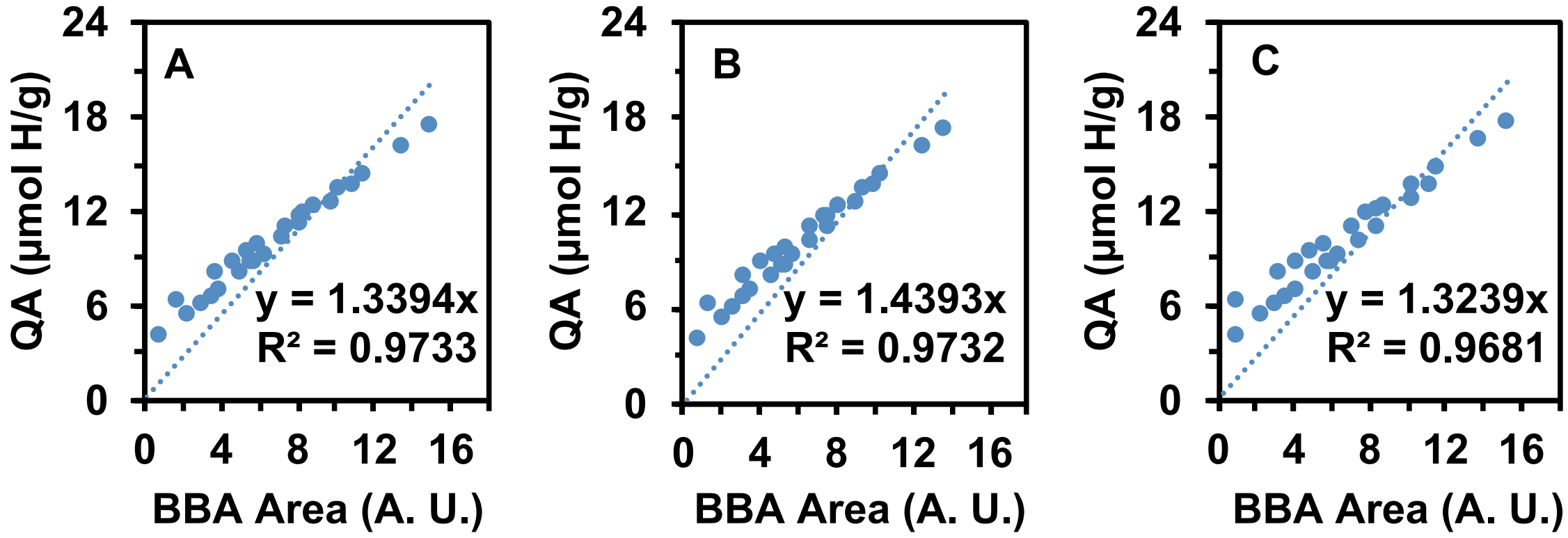

Figure S15: BBA-chemisorption calibration plots for 3 samples of 1% Au/rutile at 130 and 160 °C where the slope of each line is the calibration factor.

SI.3 Thermodynamic Calculations

Isotherms were collected by in situ FTIR spectroscopy (and calibrated as above) at four temperatures in order to perform thermodynamic analysis.

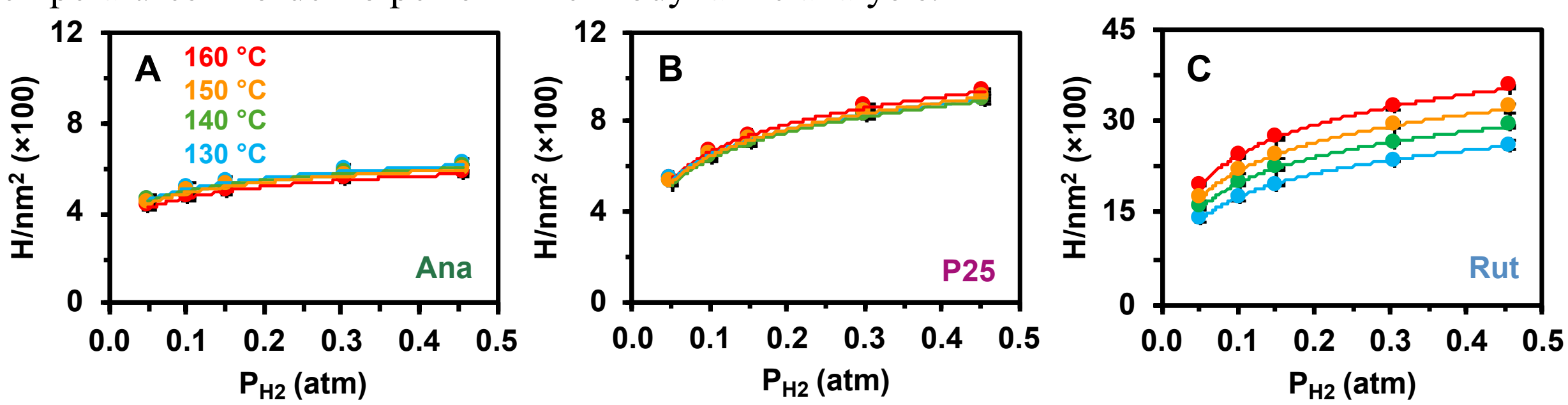

Figure S16: H_2 adsorption isotherms on 1% Au catalysts collected via in-situ FTIR spectroscopy. Panels A-C show adsorption on ana **(A)**, P25 **(B)**, and rut **(C)** at 130 °C (blue), 140 °C (green), 150 °C (orange) and 160 °C (red).

SI.3.1. K_θ Calculations

As described in our previous publication,[4] it is essential to extract adsorption equilibrium constants in a way that minimizes assumptions about surface interactions and changes. We therefore calculate K at each measured pressure (K_P) from the ratio of reactant to product activities

(**Equation S13**). We use Campbell's value for the standard state of a 2D adsorbate (1.94 x 10^{-7} mol/m^2 at 298.15K and 1 bar) to calculate product activity, as it provides the clearest entropic correlation between the 2D and 3D standard states.[14]

To extract K_p from experimental data, we first explicitly define the net gas-phase adsorption equilibrium:

$$H_{2(g)} \rightleftharpoons H_{2(ads)} \tag{S12}$$

where K_p is the ratio of activities (a) of products to reactants:

$$K_p = {a_{ads}}/{a_g} \tag{S13}$$

The activity for a 2D ideal gas is defined as the ratio of concentration (N) to the standard state concentration (N^0) for that species:

$$K_p = \frac{(N_{H_2\,(ads)}/N^0_{H_2\,(ads)})}{(N_{H_2\,(g)}/N^0_{H_2\,(g)})} \tag{S14}$$

We used Campbell's standard state concentration for $H_{2(ads)}$ (1.94 x 10^{-7} mol/m^2 at 298.15K); the standard state for $N^0_{H_2\,(g)}$ is commonly accepted as 1 bar at 298.15K.[14] The $N_{H_2\,(g)}$ is treated as the experimental P_{H2} in bar. Note the adsorbate is represented as H_2 rather than the experimentally measured H*. These two representations are analogous at this stage of the analysis, but describing the adsorbate as H_2 adheres to Campbell's standard state for non-dissociative adsorption and further minimizes entropic assumptions within K_p.[14]

Evaluating adsorption equilibria at constant coverage is far more critical for entropically driven systems than for other systems, as even small changes in coverage can have a large impact on determined thermodynamic properties. Therefore, quantitative comparisons between different temperatures requires evaluating the equilibrium constants at identical H* coverage.

Defining a H* coverage to examine prior to or while carrying out an adsorption experiment is not practically feasible, so we used this treatment to develop a continuous fitted function of K_p versus H* coverage from each isotherm. These functions have no direct physical interpretation, but they accurately describe how K_p changes with H*. These fitted functions can, therefore, be used to determine the adsorption equilibrium constant at any surface concentration within the H* concentration range provided in the measurements.

Figure S17 shows the determined K_p versus H* functions for all the adsorption data. We refer to the K_p values evaluated at this coverage as $K_{\theta,}$ but highlight all K_θ values are essentially determined directly from the full adsorption isotherm. Van't Hoff analyses of the K_θ values (see below) ensure constant H* translational entropy and minimize entropy-enthalpy compensation effects.

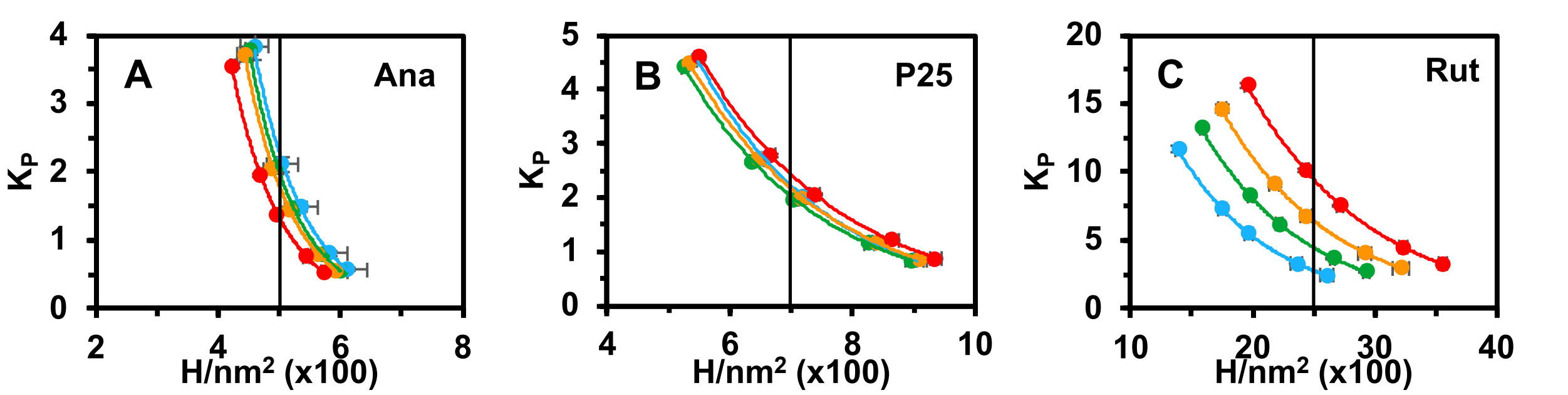

Figure S17: Equilibrium constant values calculated at each H_2 coverage, measured in the FTIR, for each isotherm collected. The vertical line represents an example H_2 coverage used to extract K_θ values used for van't Hoff analysis below.

SI.3.2. Van't Hoff Analysis

Extracted equilibrium constants were used to perform van't Hoff analyses at four coverages for each catalyst (**Figure S18**). By plotting Ln(K) vs 1000/RT, we use the van't Hoff equation (**Equation S15**) to calculate apparent ΔH and ΔS values.

$$Ln(K_\theta) = \frac{-\Delta H_{app}}{RT} + \frac{\Delta S_{app}}{R} \tag{S15}$$

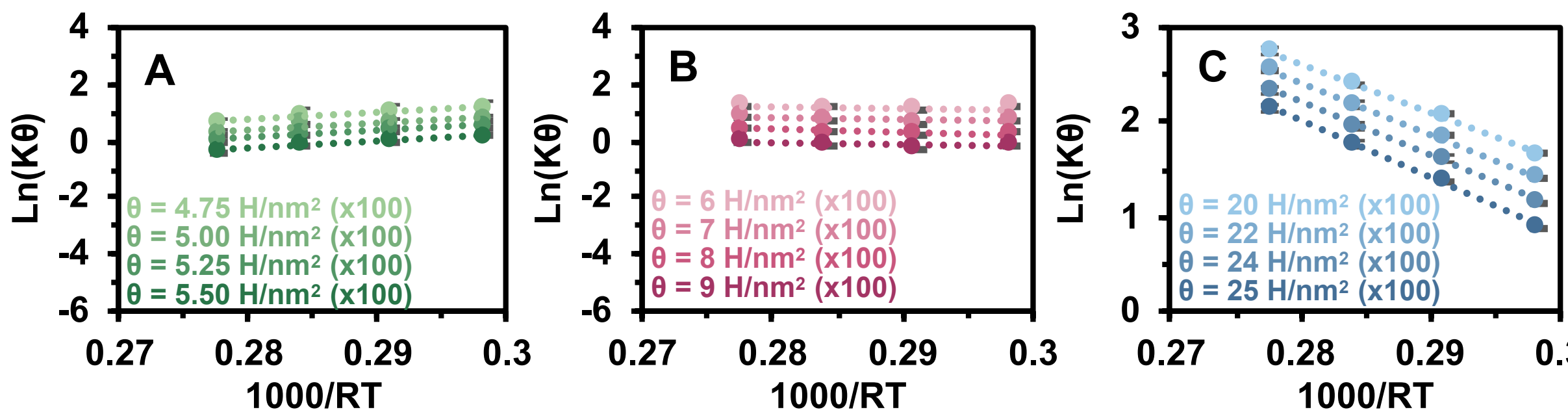

Figure S18: Van't Hoff plots of four different coverages for H_2 adsorption on 1% Au/anatase **(A)**, 1% Au/P25 **(B)**, and 1% Au/rutile **(C)**.

<u>SI.4. References</u>

(1) Zhu, S.; Gorski, W.; Powell, D. R.; Walmsley, J. A. Synthesis, structures, and electrochemistry of gold(III) ethylenediamine complexes and interactions with guanosine 5'-monophosphate. *Inorg. Chem*. 2006, 45 (6), 2688–2694.

(2) Regalbuto, J. R.; Navada, A.; Shadid, S.; Bricker, M. L.; Chen, Q. an experimental verification of the physical nature of Pt adsorption onto alumina. *J. Catal.* 1999, 184 (2), 335–348.

(3) Mahdavi-Shakib, A.; Whittaker, T. N.; Yun, T. Y.; Sravan Kumar, K. B.; Rich, L. C.; Wang, S.; Rioux, R. M.; Grabow, L. C.; Chandler, B. D. The role of surface hydroxyls in the entropy-driven adsorption and spillover of H2 on Au/TiO2 catalysts. *Nat. Catal.* 2023, 6 (8), 710–719.

(4) Hart, K. D.; Hollobaugh, M. J.; Battiste, A. M.; Yun, T. Y.; Abraham, A. P.; Hamidizirasefi, M.; Loscher, I. M.; Chandler, B. D. Upside-down adsorption: the counterintuitive influences of surface entropy and surface hydroxyl density on hydrogen spillover. *J. Am. Chem. Soc.* 2024, 146 (44), 30091–30103.

(5) Yun, T. Y.; Chandler, B. D. surface hydroxyl chemistry of titania- and alumina-based supports: quantitative titration and temperature dependence of surface brønsted acid–base parameters. *ACS Appl. Mater. Interfaces* 2023, 15 (5), 6868–6876.

(6) Cárdenas-Lizana, F.; Gómez-Quero, S.; Baddeley, C. J.; Keane, M. A. tunable gas phase hydrogenation of m-dinitrobenzene over alumina supported Au and Au–Ni. *Appl. Catal. A: General* 2010, 387 (1), 155–165.

(7) Jia, J.; Haraki, K.; Kondo, J. N.; Domen, K.; Tamaru, K. selective hydrogenation of acetylene over Au/Al2O3 catalyst. *J. Phys. Chem. B* 2000, 104 (47), 11153–11156.
(8) Cárdenas-Lizana, F.; Gómez-Quero, S.; Jacobs, G.; Ji, Y.; Davis, B. H.; Kiwi-Minsker, L.; Keane, M. A. Alumina supported Au–Ni: surface synergism in the gas phase hydrogenation of nitro-compounds. *J. Phys. Chem. C* 2012, 116 (20), 11166–11180.
(9) Kwon, S.; Lin, T. C.; Iglesia, E. formic acid dehydration rates and elementary steps on lewis acid–base site pairs at anatase and rutile TiO2 surfaces. *J. Phys. Chem. C* 2020, 124 (37), 20161–20174.
(10) Norton, P. R.; Richards, P. J. The heat of adsorption of hydrogen on platinum. *Surf. Sci.* 1974, 44 (1), 129–140.
(11) Yun, T. Y.; Battiste, A. M.; Pathickal Abraham, A.; Hart, K. D.; Chandler, B. D. Surface entropy mediated hydrogen spillover on Au/TiO2: influences of strongly adsorbed water on h2 adsorption thermodynamics. *J. Am. Chem. Soc*. 2025, 147 (33), 29908–29918.
(12) Mahdavi-Shakib, A.; Kumar, K. B. S.; Whittaker, T. N.; Xie, T.; Grabow, L. C.; Rioux, R. M.; Chandler, B. D. Kinetics of H2 adsorption at the metal–support interface of Au/TiO2 catalysts probed by broad background IR absorbance. *Angew. Chem. Int. Ed*. 2021, 60 (14), 7735–7743.
(13) Mahdavi-Shakib, A.; Rich, L. C.; Whittaker, T. N.; Chandler, B. D. Hydrogen adsorption at the Au/TiO2 interface: quantitative determination and spectroscopic signature of the reactive interface hydroxyl groups at the active site. *ACS Catal*. 2021, 11 (24), 15194–15202.
(14) Campbell, C. T.; Sprowl, L. H.; Árnadóttir, L. equilibrium constants and rate constants for adsorbates: two-dimensional (2D) ideal gas, 2D ideal lattice gas, and ideal hindered translator models. *J. Phys. Chem. C* 2016, 120 (19), 10283–10297.
